# Dynamic phenotypic reprogramming and chemoresistance induced by lung fibroblasts in small cell lung cancer

**DOI:** 10.1038/s41598-024-52687-z

**Published:** 2024-02-05

**Authors:** Yuanhua Lu, Hui Li, Peiyan Zhao, Lin Tian, Yan Liu, XiaoDan Sun, Ying Cheng

**Affiliations:** 1 Postdoctoral Research Workstation, Jilin Cancer Hospital, Changchun, China; 2 Medical Oncology Translational Research Lab, Jilin Cancer Hospital, No. 1066, Jinhu Road, High-tech District, Changchun, 130012 Jilin China; 3 Department of 1st Gynecologic Oncology Surgery, Jilin Cancer Hospital, Changchun, China; 4 Department of Medical Thoracic Oncology, Jilin Cancer Hospital, Changchun, China

**Keywords:** Cancer, Cancer microenvironment, Lung cancer, Tumour heterogeneity, Cell biology, Cell signalling

## Abstract

Small cell lung cancer (SCLC) is heterogenous in phenotype and microenvironment. Dynamic phenotypic reprogramming, leading to heterogeneity, is prevalent in SCLC, while the mechanisms remain incompletely understood. Cancer-associated fibroblasts (CAFs) possess comprehensive roles in cancer progression, while their function in phenotypic reprogramming of SCLC remain elusive. Here, we obtained transcriptome data of SCLC tissues from publicly available databases, subsequently estimated abundance of CAFs. We found CAF-abundant SCLC exhibited non-neuroendocrine (Non-NE) characteristics. Supporting this, the positive correlation of expression level of α-SMA, the CAF marker, and expression level of REST, protein typically expressed in Non-NE type SCLC, was identified in SCLC tissue arrays. Moreover, we revealed that fibroblasts inhibited NE markers expression and cell proliferation of SCLC cells in the co-culture system comprising lung fibroblasts and SCLC cells, indicating a phenotypic reprogramming from NE to Non-NE. During this process, fibroblast-derived IL-6 activated the JAK2/STAT3 signaling, upregulated c-MYC expression, and subsequently activated the NOTCH pathway, driving phenotypic reprogramming. Moreover, CAF-enriched SCLC exhibited increased immune cell infiltration, elevated expression of immune activation-related signatures, and checkpoint molecules. Our data also highlighted the chemoresistance induced by fibroblasts in SCLC cells, which was effectively reversed by JAK inhibitor. In conclusion, fibroblasts induced phenotypic reprogramming of SCLC cells from NE to Non-NE, likely contributes to inflamed immune microenvironment and chemoresistance. These findings provide novel insights into the clinical implications of CAFs in SCLC.

## Introduction

SCLC constituting around 15% of lung cancer cases, is a highly aggressive malignancy known for its rapid growth and early metastasis^1^. While initial chemotherapy often yields strong responses in extensive-stage SCLC patients, majority of them acquired resistance and relapse within a year^2^. Immune checkpoint blockade (ICB), involving anti-programmed cell death protein 1 (PD-1) or anti-programmed death-ligand 1 (PD-L1), have transformed SCLC treatment after decades of limited progress, the addition of ICB to chemotherapy as the first-line therapy prolonged the overall survival (OS) and progression-free survival (PFS) in SCLC patients^3^. However, given the strong heterogeneity in SCLC, only small group of patients acquired transformative benefits from ICB, but as a whole, ICB benefits are minimal^4^. For this, an in-depth comprehension of SCLC heterogeneity and its formation mechanism is urgently needed.

SCLC is marked by substantial heterogeneity in both tumor phenotype and microenvironment. While most SCLCs exhibit a high-grade neuroendocrine (NE) phenotype, some assume a Non-NE phenotype due to limited expression of NE markers like achaete-scute homologue 1 (ASCL1), synaptophysin (SYP), INSM transcriptional repressor 1 (INSM1), neural cell adhesion molecule 1(NCAM1), etc.^5,6^. Otherwise, four subtypes defined by the predominant transcriptional regulatory patterns based on the expression of ASCL1, neurogenic differentiation factor 1 (NEUROD1), POU class 2 homeobox 3 (POU2F3), and yes-associated protein 1 (YAP1) have reached consensus^7^. SCLC-A and SCLC-N represent classic NE phenotypes, while SCLC-P and SCLC-Y manifest as Non-NE phenotypes. Non-NE SCLC display heightened immune cell infiltration and immune checkpoint molecule expression compared to NE SCLC^8^. Moreover, SCLC-Y with upregulated interferon-γ response genes, 18-gene T-cell–inflamed gene expression profile (T-cell-inflamed GEP) score, HLA and T-cell receptor genes^9^, and greater benefits from immune checkpoint blockade (ICB) treatment^10^. These studies suggest a close correlation between subtypes and microenvironment in SCLC. Like most cancers, Phenotypic plasticity is a hallmark of SCLC, marked by a transition from NE to Non-NE phenotypes^11,12^, evidenced by reduced NE marker expression and cell proliferation^7,13–15^. Although previous studies identified determinants of phenotypic reprogramming, such as NOTCH pathway activation and overexpression of c-MYC, YAP1, RUNX2 or ZFP36L1^13–15^, regulators of phenotypic plasticity, especially those in the microenvironment, remain incompletely understood.

As the prominent cell fraction in tumor microenvironment, CAFs play diverse roles in tumor progression, therapeutic resistance, and immune modulation^16^. Their involvement in reprograming cancer cell phenotype has been validated in multiple cancers including SCLC, where they impact cancer cell stemness and epithelial-to-mesenchymal (EMT) plasticity^17,18^. However, the function of CAFs in NE phenotypic reprogramming, to our knowledge, has not been reported. Otherwise, CAFs modulate immune response via interacting with immune cells or exercise immune related functions by oneself, like antigen presentation^19–21^. The Antigen-Presenting CAFs (apCAF) have been identified in pancreatic ductal adenocarcinoma^22^. Additionally, CAFs have been reported to associated with higher expression of checkpoint molecules, in their own surface or to upregulate checkpoint molecules on other cells^23–25^. Moreover, the role of CAFs in chemoresistance has also been prevalently reported^26,27^. IL-6 is an important mediator of cross-talk between CAF and tumor cell, CAF derived IL-6 can regulate immune response, drug resistance and tumor metastasis in multiple cancers^28,29^. However, these findings primarily stem from other cancer types, with limited insight into CAF function in SCLC. Given the comprehensive function of CAFs, a deeper understanding of CAF influence on SCLC progress is imperative.

In this study, we demonstrated the association between CAFs and Non-NE phenotype in SCLC tissue samples, subsequently revealed that SCLC cells undergo phenotypic reprogramming from NE to Non-NE upon co-culture with fibroblasts, as NE markers were down-regulated and cell proliferation was suppressed in co-cultured SCLC cells. Within this process, fibroblast-derived IL-6 orchestrated JAK2/STAT3/c-MYC/NOTCH axis to drive phenotypic reprogramming of SCLC cells. Furthermore, SCLC with robust abundance of CAFs with inflamed immune microenvironment and chemoresistance, while JAK inhibitor reversed chemoresistance induced by fibroblasts. This work shed light on novel mechanism of phenotypic reprogramming, and expanded understanding of CAFs in SCLC.

## Results

### CAF-abundant SCLC exhibited Non-NE phenotype characteristics

**Figure 1:**
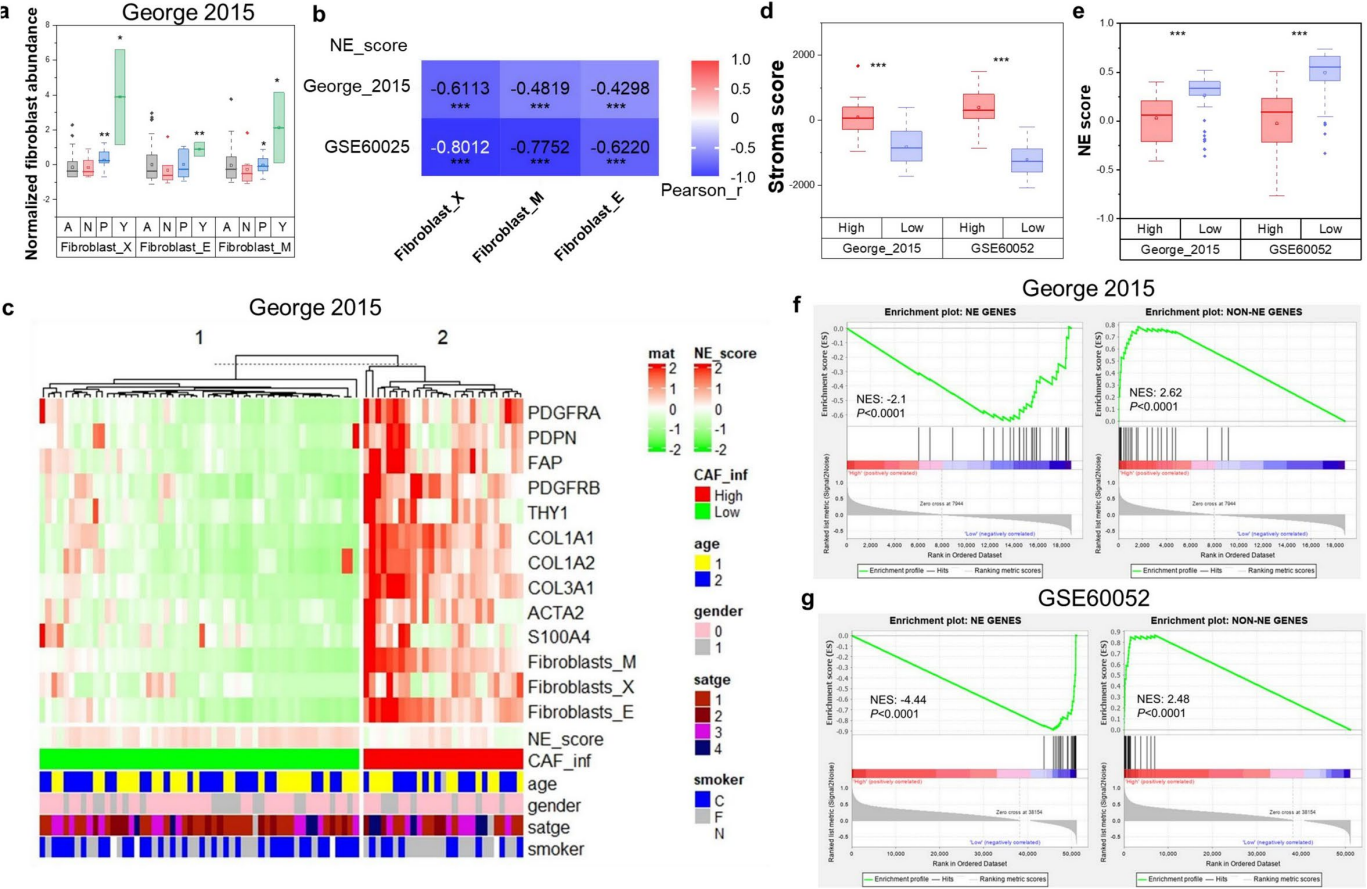
CAFs associated with Non-NE phenotype in clinical SCLC samples. (**a**) CAF abundance of SCLC-A/N/P/Y subtypes in George’s cohort (n = 81); (**b**) Pearson’s correlation of NE-score with CAF abundance in George’s cohort and GSE60052 dataset (n = 79); (**c**) Heatmap displayed the expression of CAF markers and abundance of CAFs and clustering of CAF infiltration in George’s SCLC cohort, NE scores and clinical information including age (1: < 65-year-old, 2: ≥ 65-year-old), gender (0: female, 1: male), stage (UICC stage) and smoker (C: current, F: former, N: never) were presented under the heatmap; (**d**) The Stroma score estimated by ESTIMATE of CAF high- and low-infiltration groups in George’s cohort and GSE60052 dataset; (**e**) NE score of CAF high- and low-infiltration groups in George’s cohort and GSE60052 dataset; (**f**,**g**) GSEA with normalized enrichment score (NES) and nominal P-values for NE and Non-NE gene lists in CAF high- and low-infiltration group in George’s cohort (**f**) and GSE60052 dataset (**g**). ***P < 0.001.

To gain insights into the heterogeneity of CAF abundance across SCLC transcriptional subtypes, xCell, MCP-counter and EPIC algorithms were employed to estimate the CAF abundance in RNA-seq data of SCLC tissues, including both George’s RNA-seq cohort and GSE60052 dataset. SCLC-A/N/P/Y subtypes within Geroge’s cohort were identified using ASCL1, NEOROD1, POU2F3, and YAP1 expressions as previous reported^7^ (Fig. S1a). In our results, the SCLC-Y and SCLC-P subtypes exhibited higher CAF abundance than SCLC-A and SCLC-N subtypes (Fig. 1a). Moreover, a significant negative correlation between CAF abundance and NE score was evident (Fig. 1b). To further confirm the association between CAFs and NE phenotype, we employed CAF markers and abundance-based classifier identified two distinct CAF infiltration clusters in SCLC cohorts (Fig. 1c, Fig. S1b). Subtype distributions, NE scores and patients clinical features (age, gender, stage and smoking status) were displayed under the heatmap (Fig. 1c). As data shown, less than 30% of SCLC patients with high infiltration of CAFs (24/81 in George’s cohort and 20/79 in GSE60052) (Fig. 1c, Fig. S1b). The presence of significant higher stroma score in high-infiltration group further validated the clustering results (Fig. 1d). Additionally, NE scores of CAF high-infiltration group were significantly lower than that in the low-infiltration group in both George’s cohort and GSE60052 (Fig. 1e), and the NE gene list from Zhang et al.^62^ showed down-regulation in the high CAF infiltration group (NES: − 2.1, − 4.44, *P* < 0.0001), while the Non-NE gene list was up-regulated in the same group (NES: 2.62, 2.48, *P* < 0.0001) (Fig. 1f,g). These data suggested CAF abundance was heterogeneity across different subtypes and SCLC with abundant CAFs showed Non-NE characteristics.

### Expression of α-SMA in SCLC tissue was positively correlated with Non-NE marker

**Figure 2:**
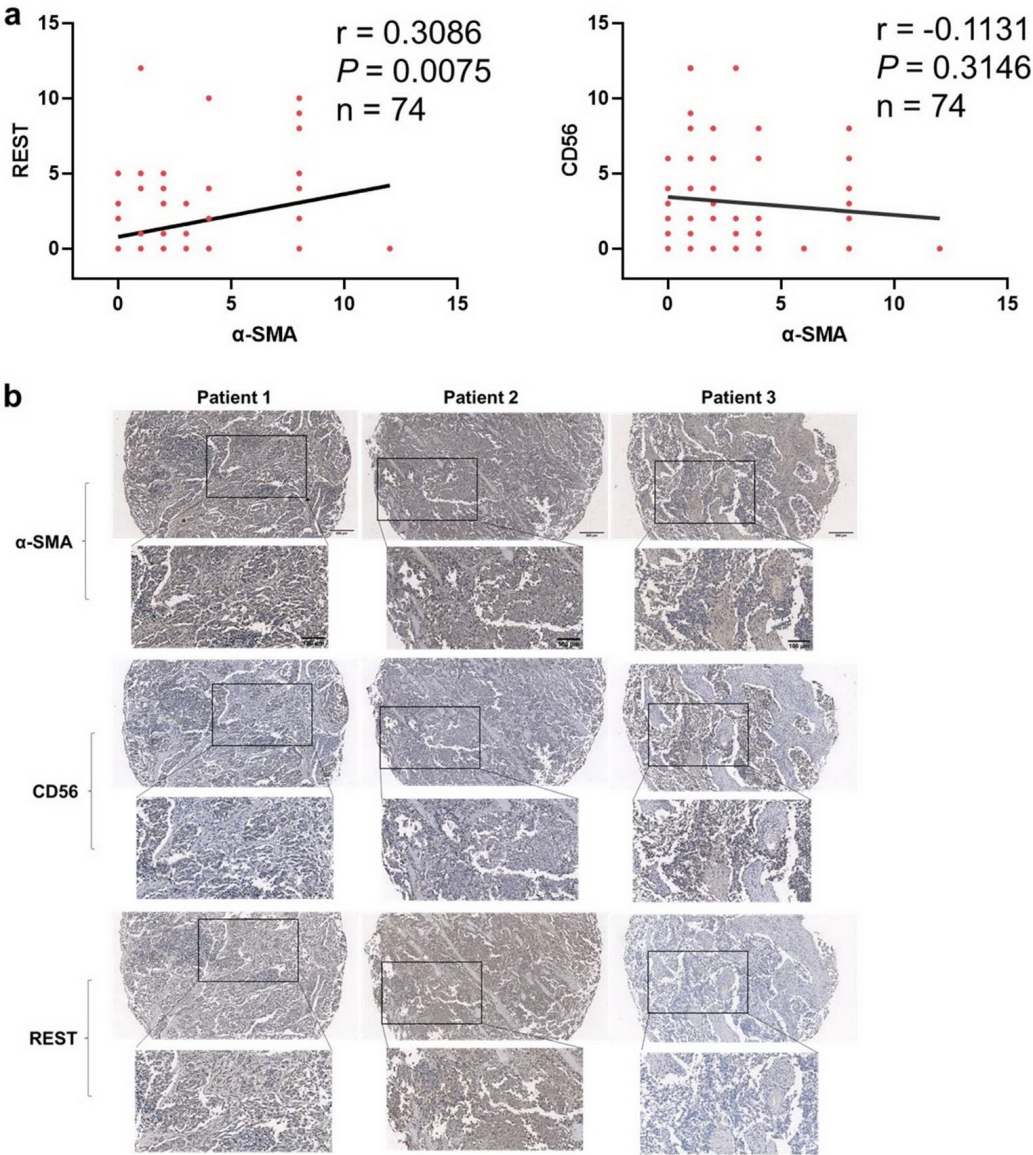
Expression of CAF marker was correlated with Non-NE marker in SCLC tissues. (**a**) Pearson’s correlation of α-SMA expression level with CD56 expression level and REST expression level (n = 74); (**b**) IHC staining of SCLC tissue arrays was performed to evaluate the expression of α-SMA, CD56 and REST.

To validate the correlation of CAF and Non-NE phenotype in SCLC, IHC staining was performed in SCLC tissue arrays to evaluate the expression of α-SMA, the CAF marker, and REST, the target of NOTCH pathway, commonly expressed in Non-NE type of SCLC^6^. As data shown, the expression of α-SMA was significantly positively correlated with REST expression (Pearson r = 0.3086, *P* = 0.0075) (Fig. 2a,b). Expression of CD56, the NE marker, trended towards to negative correlation with α-SMA expression, though statistically it makes no difference (Pearson r = − 0.1131, *P* = 0.3146) (Fig. 2a,b). These findings further confirmed the correlation of CAF with Non-NE phenotype of SCLC.

### Fibroblasts contributed to the reprogramming of SCLC cells from NE to Non-NE phenotype via IL-6

**Figure 3:**
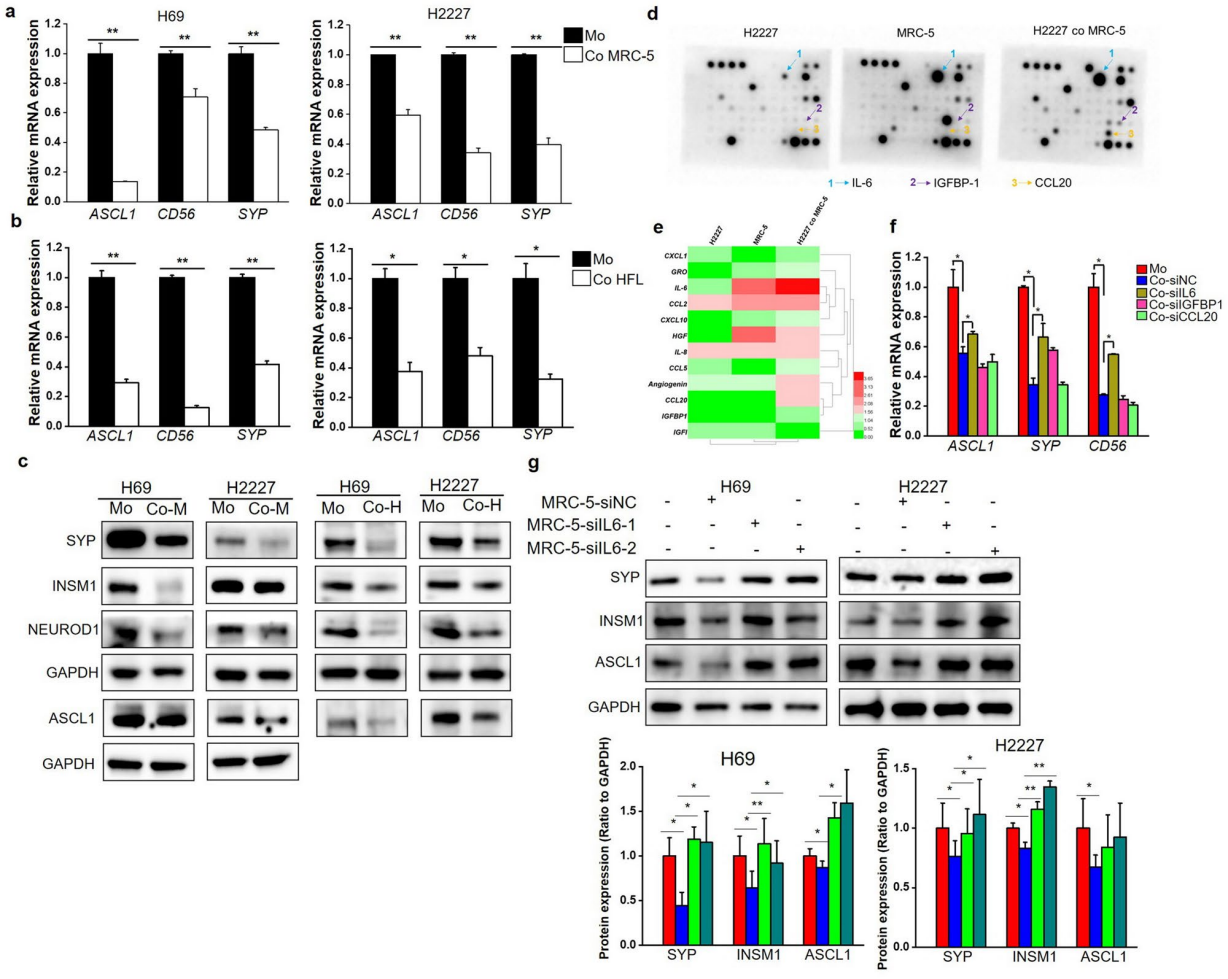
Fibroblast-derived IL-6 promoted phenotypic reprogramming of SCLC cells. (**a**,**b**) H69 and H2227 cells co-cultured with MRC-5 cells (**a**) or HFL1 (**b**) respectively and the expression of NE genes in H69 and H2227 cells was measured by qRT-PCR (n = 3); (**c**) The protein expression of NE markers was determined by Western blot analysis and the blots were cropped prior to hybridisation with antibodies, original images were displayed in Supplementary information. (**d**,**e**) Antibody arrays were performed in serum-free culture medium from separately cultured or co-cultured H2227 and MRC-5 cells, membranes were scanned by chemiluminescence instrument (**d**) and quantified by image J software, the levels of cytokines were normalized to positive controls (**e**); (**f**) The expression of NE genes in H2227 cells cultured separately or co-cultured with MRC-5 cells which transfected with siRNA targeted to IL6, CCL20 or IGFBP1 or negative control were determined by qRT-PCR (n = 3); (**g**) Western blot was used to analyze the expression of NE markers in H69 and H2227 cells which cultured separately or co-cultured with MRC-5 cells which transfected with siRNA targeted to IL6 or negative control, the blots were cropped prior to hybridisation with antibodies, original images were displayed in Supplementary information. Quantification of blots were performed using Image J software (n = 2). *P < 0.05, **P < 0.01.

The reprogramming from NE to Non-NE phenotype has been widely reported in SCLC. To confirm whether the association of CAFs with Non-NE phenotype is involve in phenotypic reprogramming, we established a co-culture system comprising lung fibroblasts and SCLC cells. Results demonstrated that co-culturing with MRC-5 or HFL1 fibroblasts by inserts effectively suppressed NE genes in NE-type SCLC cells (H69 and H2227 cells) (Fig. S2a, Fig. 3a,b). These downregulated genes encompassed ASCL1 (a critical transcription factor for the NE phenotype), NCAM1 (a membrane marker of NE cells), and SYP (encoding functional protein for NE cells). Additionally, the protein expression of NE markers in co-cultured SCLC cells was reduced compared to SCLC cells cultured alone (Fig. 3c). Furthermore, co-culture led to the inhibition of H69 cell proliferation and the clonal formation capacity of H2227 cells (Fig. S2b,c), aligning with the concept of Non-NE-type SCLC cells manifesting slower growth than NE-type cells^6^. Notably, the expression of α-SMA in mRNA level and protein level was both significantly up-regulated in MRC-5 after co-culture with H69 or H2227 cells (Fig. S2d,e), indicating that co-cultured fibroblasts acquired CAF characteristics. These results support the role of CAFin promoting reprogramming of SCLC cells to Non-NE phenotype.

We observed NE phenotypic reprogramming in SCLC cells after co-culture with fibroblasts, even in the absence of direct contact with fibroblasts, suggests a pivotal role of paracrine regulation. To identify the key fibroblast-derived regulator, we employed human cytokine antibody arrays and found elevated levels of several cytokines, particularly IL-6, in the co-culture medium of H2227 and MRC-5 cells compared to separate cultures (Fig. 3d,e). IL-6 secretion in the co-culture system was indeed higher than that of separately cultured fibroblasts or SCLC cells (Fig. S3a). To investigate the specific role of IL-6, we inhibited the expression of candidate cytokines, including IL-6, IGFBP1, and CCL20, in MRC-5 cells (Fig. S3b). Encouragingly, inhibiting IL-6 expression in MRC-5 cells partially restored the diminished expression of NE genes in H2227 cells induced by MRC-5 cells (Fig. 3f). Furthermore, IL6 and IL6R levels were significantly up-regulated in fibroblasts and SCLC cell lines, respectively, following co-culture (Fig. S3c,d). Correspondingly, NE marker protein expression in H69 and H2227 cells, impeded by MRC-5 cells, rebounded upon IL-6 knockdown in MRC-5 cells (Fig. 3g). Moreover, the inhibitory effect of HLF1 cells on H69 and H2227 cell proliferation was diminished upon IL6 knockdown in HLF1 cells (Fig. S3e). These data indicated the critical role of fibroblast-derived IL-6 in phenotypic reprogramming of SCLC cells. In clinical context, a significant negative correlation between IL6 expression and NE score surfaced in both George’s cohort and GSE60052, which further substantiated the potential role of IL-6 in phenotypic reprogramming from NE to Non-NE. Moreover, IL6 expression positively correlated with CAF abundance (Fig. S3f), implying CAFs as significant IL-6 sources in SCLC.

### Fibroblasts activated JAK2/STAT3 and NOTCH pathways in SCLC cells

**Figure 4:**
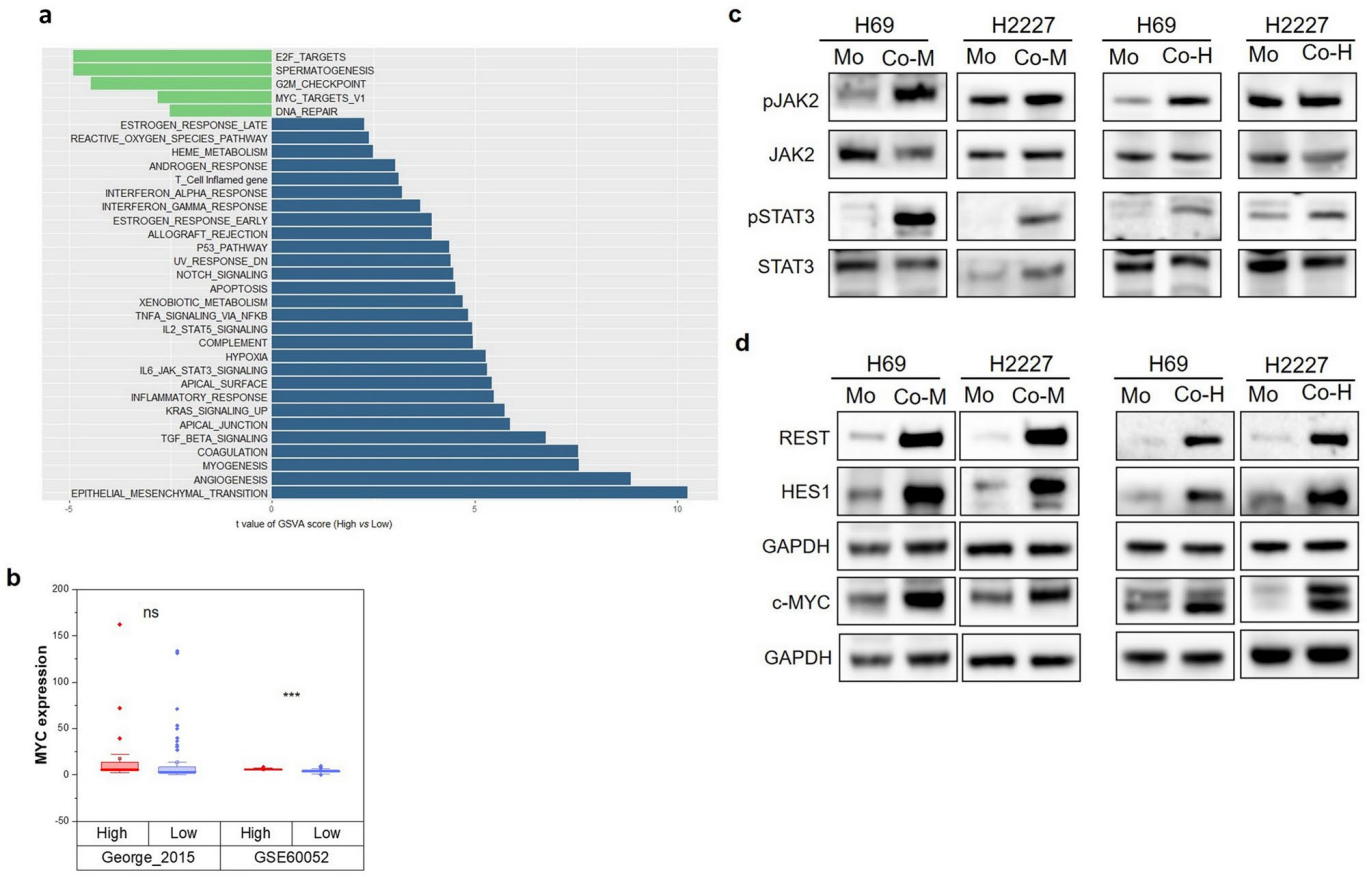
JAK2/STAT3 signaling and NOTCH signaling were activated in co-culture system. (**a**) Bar chart of t value of GSVA score between high- and low-infiltration group in George’ cohort, the blue bars represent hallmarks significantly enriched in high-infiltration group and green bars means hallmarks significantly enriched in low-infiltration group (*P* < 0.05); (**b**) MYC expression of CAF high- and low-infiltration groups in George’s cohort and GSE60052 dataset; (**c**,**d**) The expression of total and phosphorylated JAK2 (Tyr1007), STAT3 (Ser727) (**c**) as well as c-MYC, REST and HES1 (**d**) in H69 and H2227 cells with or without HFL1 were determined by western blot analysis, the blots were cropped prior to hybridisation with antibodies, original images were displayed in Supplementary information.

To explore the underlying mechanism of fibroblast-induced phenotypic reprogramming, we compared hallmark-signaling pathways in cancer using GSVA scores between the CAF high-infiltration group and the low-infiltration group. The most significant enrichment pathway in the CAF high-infiltration group was epithelial-mesenchymal transition (Fig. 4a). Given that the NE phenotype presents epithelial traits, while the Non-NE phenotype showcases mesenchymal features, this result further confirmed the role of CAFs in phenotypic reprogramming from NE to Non-NE. Otherwise, IL-6/JAK/STAT3 signaling and NOTCH signaling significantly enriched in the CAF high-infiltration group (Fig. 4a), the heightened expression of MYC was also observed in the CAF high-infiltration group (Fig. 4b). Given the results described above that IL-6 was the critical mediator in fibroblast-caused phenotypic reprogramming, we sought to investigate whether co-cultured with fibroblasts activate the JAK/STAT3 pathway in SCLC cells. As we expected, elevated phosphorylated levels of JAK2 and STAT3 were observed in SCLC cells upon co-culture with fibroblasts (Fig. 4c). NOTCH signaling has been proven to play an essential role in phenotypic reprogramming in SCLC, with c-MYC acting as the key regulator upstream of the NOTCH pathway^6^. Our data demonstrated that c-MYC and targets of NOTCH signaling (HES1 and REST) were upregulated in co-cultured SCLC cells (Fig. 4d), suggesting that fibroblasts activated the NOTCH signaling in SCLC cells.

### JAK2/STAT3 signaling and c-MYC mediated Fibroblast-caused phenotypic reprogramming of SCLC cells

**Figure 5:**
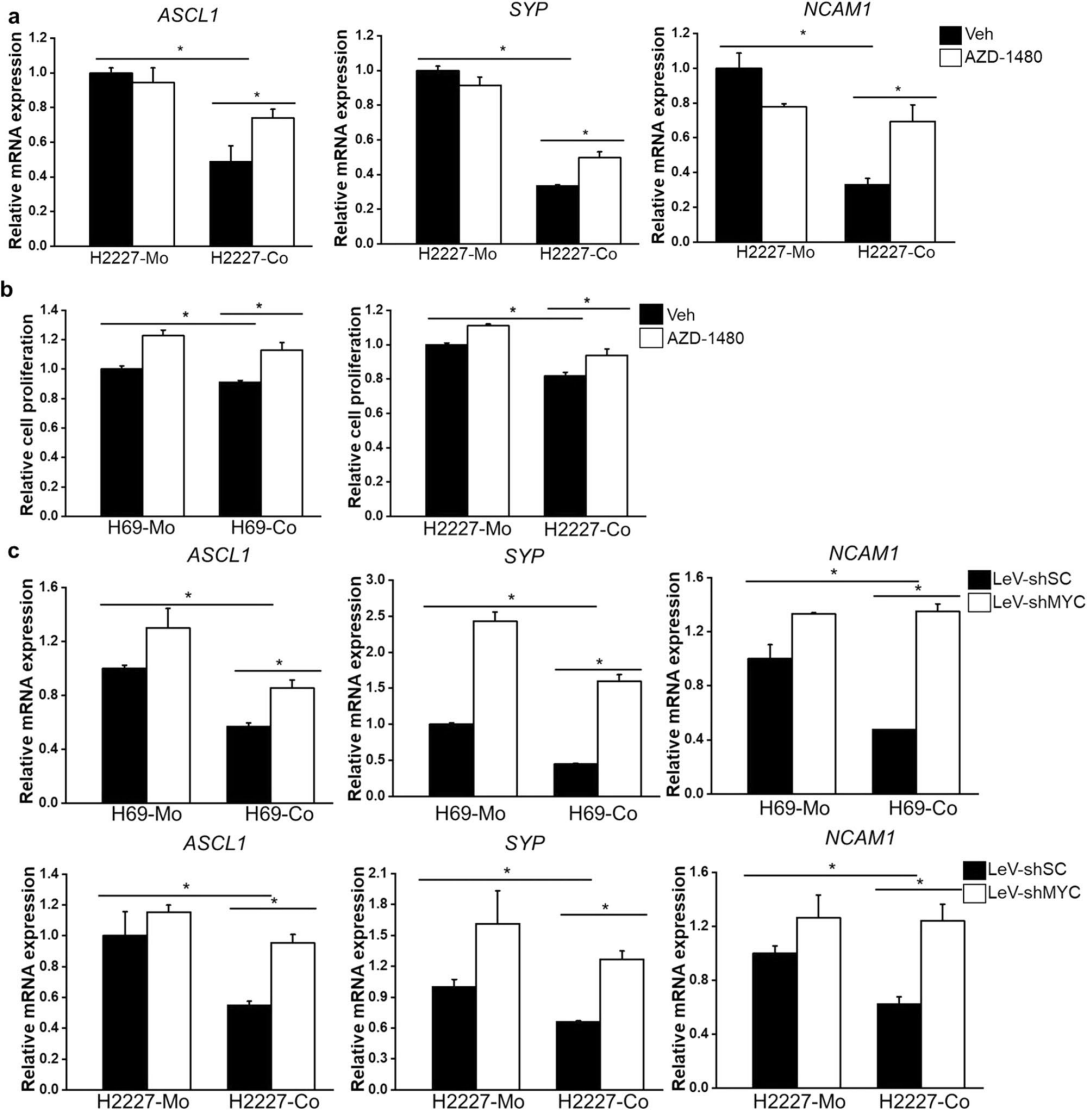
Exploration of JAK/STAT3 pathway and c-MYC in mediating CAF-induced NE phenotypic reprogramming. (**a**) QRT-PCR was used to analyze expression of NE genes in H2227 cells which pretreated with AZD-1480 and further cultured with or without HFL1 (n = 3); (**b**) Cell proliferation was determined by CCK-8 assay after stimulated with AZD-1480 (0.8 μM); (**c**) H69 and H2227 cells were infected with lentivirus incorporating MYC RNAi or scramble fragments and further cultured with or without HFL1, the expression of NE genes were determined by qRT-PCR (n = 3). *P < 0.05.

To confirm whether fibroblasts promote phenotypic reprogramming of SCLC cells dependent on JAK2/STAT3 signaling and c-MYC, the JAK2/STAT3 signaling was inhibited by AZD-1480 in the context of co-culture system and then NE phenotypic characteristics was determined. Encouraging, inhibiting the JAK2/STAT3 pathway using the AZD-1480 inhibitor restored the decreased NE gene expression in H2227 cells and attenuated the inhibitory effect of HFL1 cells on H69 and H2227 cell proliferation (Fig. 5a,b). Moreover, MYC knockdown in SCLC cells alleviated HFL1 cell-induced suppression of NE genes (Fig. 5c). These data suggested the pivotal role of JAK2/STAT3 signaling pathway and c-MYC in orchestrating fibroblast-induced phenotypic reprogramming of SCLC cells.

### Fibroblasts driven phenotypic reprogramming of SCLC cells dependent on IL-6/STAT3/c-MYC/NOTCH axis

**Figure 6:**
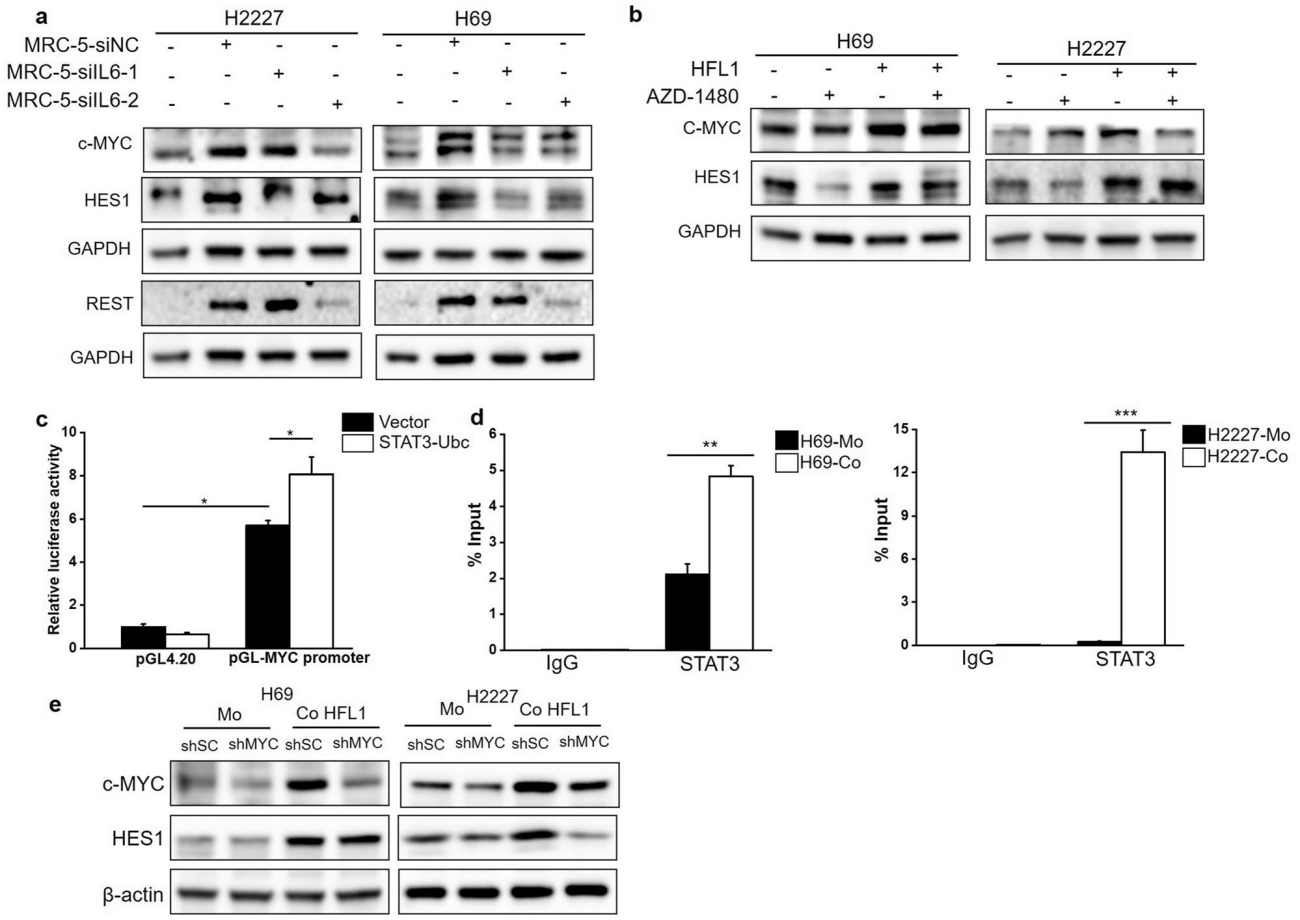
The involvement of IL-6/JAK2/STAT3/c-MYC/NOTCH axis in CAF-induced NE phenotypic reprogramming. (**a**) Western blot was used to determine the expression of c-MYC and targets of NOTCH pathway in H69 and H2227 cells which cultured separately or co-cultured with IL6 siRNA transfected MRC-5 cells, the blots were cropped prior to hybridisation with antibodies, original images were displayed in Supplementary information; (**b**) Western blot was used to analyze expression of c-MYC and HES1 in H69 and H2227 cells which pretreated with AZD-1480 and further cultured with or without HFL1 and the blots were cropped prior to hybridisation with antibodies, original images were displayed in Supplementary information; (**c**) Dual luciferase reporter analysis of MYC promoter was performed. The luciferase activity of constructs with or without MYC promoter (− 900/+ 100) in HEK-293T cells upon STAT3-Ubc plasmid or vector transient transfection was detected by fluorescence microplate reader (n = 3); (**d**) The specific region (− 628/− 503) of MYC promoter was detected by ChIP-qPCR after STAT3 or IgG immunoprecipitation in H69 and H2227 cells upon separately cultured or co cultured with HFL1 cells (n = 3); (**e**) H69 and H2227 cells were infected with lentivirus incorporating MYC RNAi or scramble fragments and further cultured with or without HFL1, the expression c-MYC and HES1 were detected by western blot analysis and the blots were cropped prior to hybridisation with antibodies, original images were displayed in Supplementary information.

As described above, IL-6, JAK2/STAT3 signaling, c-MYC, and NOTCH signaling were all found to be involved in fibroblast-induced phenotypic reprogramming. To explore the correlation among these factors during fibroblast-induced phenotypic reprogramming, we initially examined the role of fibroblast-derived IL-6 in c-MYC and NOTCH pathway. The results demonstrated that IL-6 inhibition in MRC-5 cells dampened c-MYC, REST, and HES1 elevation in co-cultured H69 and H2227 cells (Fig. 6a). Similar attenuation occurred when inhibiting the JAK2/STAT3 pathway by AZD-1480 (Fig. 6b), indicating the role of IL-6/JAK2/STAT3 signaling in up-regulating c-MYC and activating NOTCH pathway during co-culture of SCLC cells and fibroblasts. Importantly, dual-luciferase reporter assays showed that constructs containing MYC promoter fragments activated luciferase activity, and this activity was significantly enhanced upon transfection with the STAT3-Ubc plasmid, which constitutively activates STAT3 (Fig. 6c). Additionally, ChIP-qPCR assays revealed that STAT3 bound to the MYC promoter in H69 and H2227 cells, particularly after co-culture with HFL1 cells (Fig. 6d). This revealed the role of STAT3 in governing c-MYC expression at the transcriptional level and explained the mechanism by which IL-6/JAK2/STAT3 regulates c-MYC during co-culture of SCLC cells and fibroblasts. Moreover, MYC knockdown confined NOTCH pathway activation in response to HFL1 cell stimulation, evident by HES1 downregulation (Fig. 6e), which confirmed c-MYC’s role as upstream regulators of NOTCH pathway. In all, fibroblast-derived IL-6 reprogrammed SCLC cells to Non-NE phenotype dependent on the JAK2/STAT3/c-MYC/NOTCH axis.

### CAFs were associated with the inflamed microenvironment in SCLC

**Figure 7:**
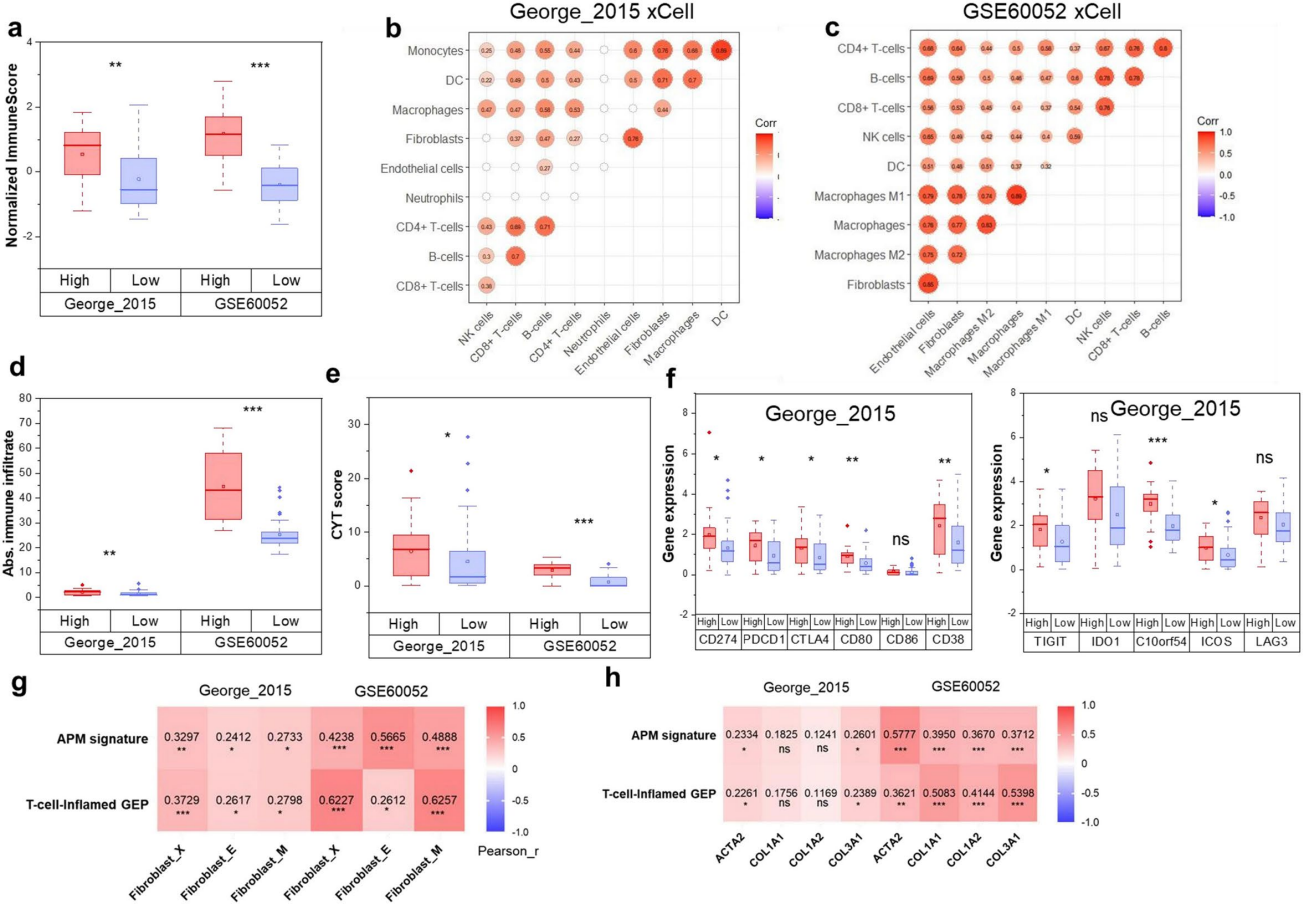
The association of CAFs with immune features in SCLC. The Immune score estimated by ESTIMATE (**a**), the Cytolytic Activity (CYT) score (**e**) and the expression value of checkpoint molecules (**f**) of CAF high- and low-infiltration groups in George’s cohort and GSE60052 dataset; (**b**,**c**) Pearson’s correlation of fibroblast abundance with immune cell abundance in George’s cohort (**b**) and GSE60052 dataset (**c**), dots with statistical significance (*P* < 0.05) were displayed; (**d**) Absolute immune infiltration estimated by CIBERSORTx of high- and low-infiltration groups in George’s cohort; (**g**,**h**) Pearson’s correlation of fibroblast abundance with APM signature and T-cell-inflamed GEP (**g**), and expression of CAF markers with APM signature and T-cell-inflamed GEP (**h**) in George’s cohort and GSE60052 dataset. *ns* no significance, *P < 0.05, **P < 0.01, ***P < 0.001.

SCLC with a Non-NE phenotype is known to exhibit inflamed microenvironment^8,30^. Given the contribution of fibroblasts in development of Non-NE SCLC cells, we explored the immune features in CAF-enriched SCLC. ESTIMATE algorithm-derived immune scores were notably higher in the high CAF infiltration group compared to the low-infiltration group in both George’s cohort and GSE60052 (Fig. 7a). We further analyzed the correlation of CAF infiltration with immune cell abundance and activity, revealing a robust positive correlation between CAF abundance and various immune cells, including macrophages, monocytes, T cells, B cells, natural killer (NK) cells and dendritic cells (DCs) (Fig. 7b,c, Fig. S4a,b). Furthermore, the CAF high-infiltration group exhibited significantly elevated total immune infiltrate (Absolute immune infiltrate) estimated via CIBERSORTx (Fig. 7d) and cytolytic activity (CYT) scores in comparison to the CAF low-infiltration group (Fig. 7e).

The challenge of ICB resistance in SCLC is attributed to low expression of interferon signatures, immune checkpoints, and the weak of antigen presentation capability^31^. We examined whether CAF infiltration correlated with these mechanisms. The comparison of hallmark-signaling pathways in cancer using GSVA scores revealed significant enrichment of immune-related pathways like inflammatory response, TNF-α signaling via NFκB, complement, and interferon α/γ response in the high-infiltration group (Fig. 4a). Furthermore, both George's cohort and GSE60052 exhibited markedly higher expression of immune checkpoint molecules in the CAF high-infiltration group, encompassing CD274 (encoding PD-L1) and PDCD1 (encoding PD-1), alongside other immune checkpoint genes (Fig. 7f, Fig. S4c). Moreover, we found CAF abundance and marker expression were positively correlated with the APM signature and T-cell-inflamed GEP (Fig. 7g,h). This finding is further substantiated by significantly higher APM signature and T-cell-inflamed GEP values in the high-infiltration groups of both George’s cohort and GSE60052 (Fig. S4d,e). Thus, SCLC with abundant CAFs seem exhibits an inflamed microenvironment, indicating their potential to forecast ICB benefits.

### CAFs induced chemoresistance of SCLC cells and reversed by JAK inhibitor

**Figure 8:**
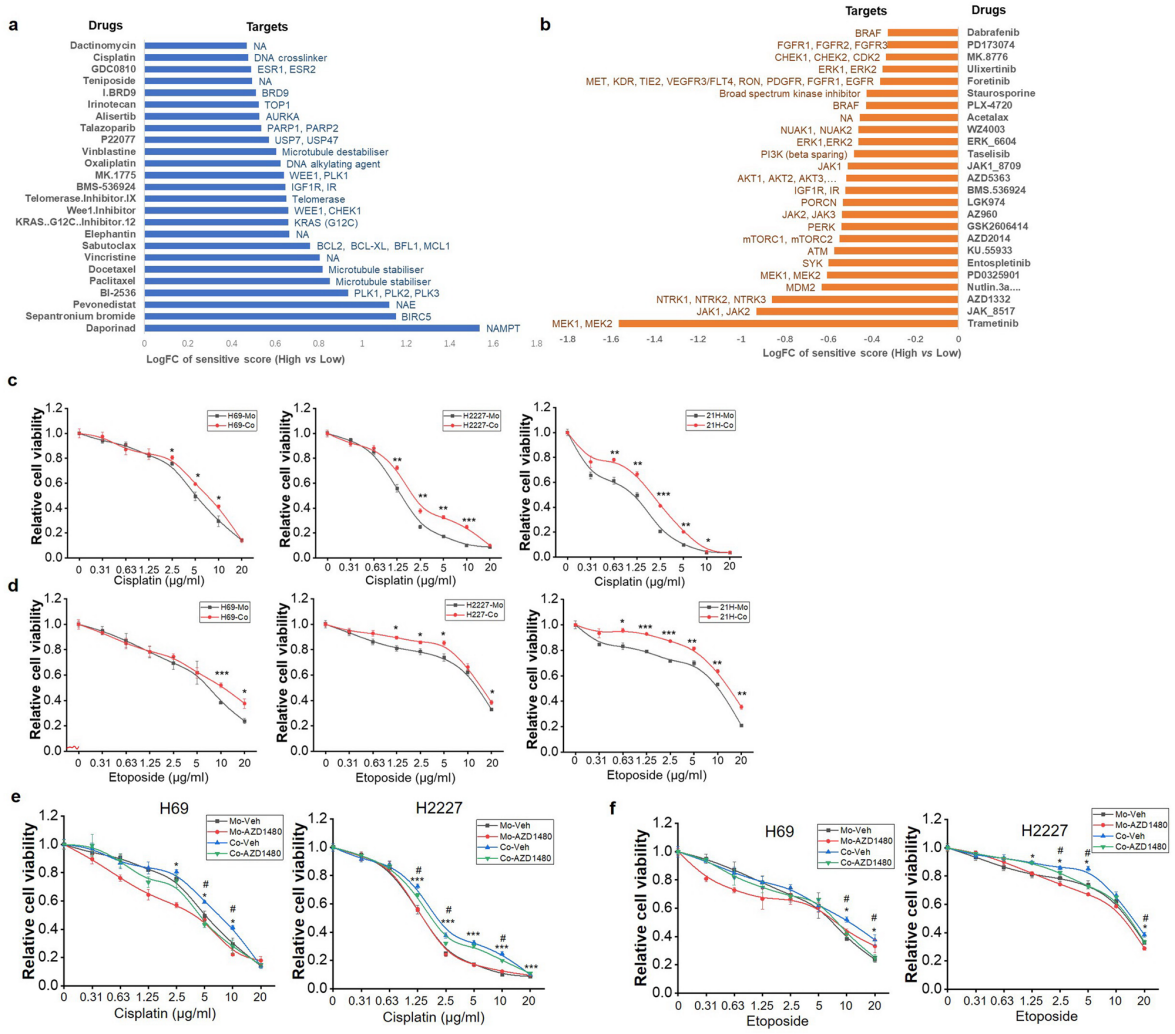
Evaluation and validation of drug sensitivity upon fibroblasts stimulation in SCLC. (**a**,**b**) The log fold change of predicted sensitivity scores of drugs based on GDSC2 database between high- and low-infiltration group in George’s cohort, Log FC > 0 means high-infiltration group is more resistant to these drugs (blue bars), Log FC < 0 represents high-infiltration group is more sensitive to these drugs (orange bars); (**c**,**d**) NE type SCLC cells were separately cultured or co-cultured with MRC-5 for 7 days, then reseeded and treated with cisplatin or etoposide for another 48 h, cell viability was detected by CCK-8 reagent (n = 3). (**e**,**f**) CCK-8 reagent was used to test the viability of separately cultured or co-cultured H69 and H2227 cells which pretreated with or without AZD-1480 and exposed to Cisplatin (**e**) or Etoposide (**f**). * or #, *P* < 0.05, ** or ##, *P* < 0.01. *, Co-Veh vs. Mo-Veh. #, Co-AZD vs. Mo-AZD.

To the previous reported critical role of CAFs in drug response of cancer cells, we assessed the influence of CAF infiltration on drug sensitivity of SCLC. The oncoPredict package was used to predict drug sensitivity from the GDSC2 database based on the gene expression profile of George’ cohort. The comparison of predicted IC50 values between high- and low-infiltration groups was conducted using the limma package. Notably, Fig. 8a presents drugs exhibiting heightened resistance within the high-infiltration group, while Fig. 8b showcases drugs evoking greater sensitivity in the same group. We found that several drug targets previously considered promising for SCLC demonstrated lower sensitivity in the high-infiltration group than low-infiltration group, including drugs targeting AURKA, PARP1/2, WEE1, and BCL2 (Fig. 8a). Conversely, increased sensitivity was noted towards certain JAK1/2/3 inhibitors within the high-infiltration group (Fig. 8a). A particularly significant observation was the reduced sensitivity to chemotherapy agents integral to the standard SCLC treatment regimen in high-infiltration group, such as Irinotecan and cisplatin (Fig. 8a). To verify these findings, mono- or co-cultured NE-type SCLC cells were exposed to chemotherapy agents Cisplatin or Etoposide—both vital components of first-line SCLC treatment. Data illustrated significantly reduced sensitivity to Cisplatin and Etoposide of co-cultured SCLC cells compared to individually cultured cells (Fig. 8c,d). This substantiated the predictive outcomes suggesting that SCLC with high CAF infiltration exhibited heightened chemotherapy resistance.

Furthermore, we explored whether JAK inhibitors could overcome the resistance of SCLC cells to chemotherapy drugs induced by fibroblasts. Co-cultured or separately cultured H69 and H2227 cells were pretreated with AZD-1480 before the treatment of Cisplatin or Etoposide. Data demonstrated that the decrease in sensitivity of SCLC cells to cisplatin and etoposide induced by fibroblasts was attenuated by AZD-1480 treatment (Fig. 8e,f). These data confirmed the predicted results in dataset and further implied that JAK inhibitors were likely to reverse the chemoresistance induced by fibroblasts.

## Discussion

Phenotypic plasticity, characterized by dedifferentiation, blocked differentiation, and trans-differentiation, is hallmark of cancer^32^, leading to heterogeneity, which also prevalent in SCLC^6,13,15^. Previous studies have primarily investigated the determinants of phenotypic remodeling from the perspective of tumor cells themselves^6,13,15,33^. However, within the intricate microenvironment, multiple components, including immune cells, stroma cells, extracellular matrix, etc., contribute to SCLC progression, while the role of microenvironmental factors in phenotypic reprogramming of SCLC is poorly understood. Additionally, the heterogeneity of CAFs, a crucial stromal cell fraction, in different subtypes has remained elusive. This work focused on the role of CAF in phenotypic reprogramming and their impact on immune features of SCLC, as well as their influence on drug response.

To explore the distribution of CAF in SCLC across different subtypes, we estimated CAF abundance by algorithms in RNA-seq data of SCLC tissues across two clinical cohorts. Importantly, CAF abundance was variable across different molecular subtypes, the significant negative correlation between CAF abundance and NE score was also observed in both two SCLC cohorts. CAF markers and abundance identified two groups with distinct CAF infiltration. The high-infiltration group constituted less than 30% in both cohorts, consistent with previous findings of limited CAF presence in SCLC^34^. Importantly, the CAF high-infiltration group exhibits significant lower NE scores than low-infiltration group, GSEA results further validated the up-regulation of Non-NE genes in high-infiltration group, within which NE genes were down-regulated. These data revealed the heterogeneity of CAF distribution cross NE and Non-NE SCLC cases and indicated the Non-NE characteristics in CAF-enriched SCLC. CAFs have been reported to contribute to cancer cell dedifferentiation and stemness^17,18^. However, their involvement in NE phenotypic reprogramming has not been reported. For the prevalent of phenotype plasticity in SCLC, we sought to explore whether CAFs involved phenotypic reprogramming of SCLC cells from NE to Non-NE. As most of SCLC patients are diagnosed in the advanced stage, they have lost the opportunity for surgery^1^. Thus, tissues for extracting primary CAFs are difficult to obtain. For this reason, a co-culture system consisting of commercially available lung fibroblasts and SCLC cells was employed in this study. Lung fibroblasts acquired CAF properties after co-cultured with SCLC cells, characterizing by increasing expression of α-SMA, which was consistent with previous reported^35,36^. Therefore, it is workable to explore the effect of CAF on SCLC cells using the co-culture system. In the co-culture context, the down-regulation of NE genes and suppression of cell proliferation induced by fibroblasts validated the role of fibroblasts in promoting NE to Non-NE phenotypic reprogramming. In this process, SCLC cell co-cultured with fibroblasts without direct contact, indicating the critical role of paracrine. We identified fibroblast-derived IL-6 was the key mediator during fibroblast-induced phenotypic reprogramming. Notably, CAFs are recognized crucial IL-6 sources in cancers^37,38^, with numerous studies emphasizing IL-6's role in cancer cell-CAF interaction^28,38^. Other cytokines may also involve in this process, but the crucial role of IL-6 definitely cannot be ignored. Otherwise, although we did not investigate whether SCLC cell direct contact with fibroblasts induced phenotypic reprogramming of SCLC cells, Nilu et al. demonstrated the EMT plasticity during SCLC cell direct co-cultured with fibroblasts^18^. Meanwhile, EMT pathway is also enriched in CAF high-infiltration group in our clinical SCLC cohort. Given the NE cells presents epithelial traits, while the Non-NE cells exhibit mesenchymal features, we speculate that NE phenotypic reprogramming will occur during SCLC cells directly contact with fibroblasts. In that case, more complex mechanisms will be involved.

Further exploration revealed enrichment of IL-6/JAK/STAT3 signaling and NOTCH signaling in CAF high-infiltration group, along with heightened expression of MYC. NOTCH signaling activation has been identified as a critical factor driving phenotypic reprogramming from NE to Non-NE, with c-MYC as a key upstream regulator^6,15^. We hypothesize that fibroblast-derived IL-6 activates the JAK/STAT3 signaling, subsequently triggering the c-MYC/NOCTH pathway to drive NE phenotypic reprogramming. Our results demonstrate that IL-6 from fibroblasts indeed activates the JAK2/STAT3 pathway in SCLC cells. Phosphorylated STAT3 binds to and upregulates MYC expression upon fibroblast stimulation. Moreover, we observed the activation of the NOTCH pathway in fibroblast-stimulated SCLC cells. Importantly, c-MYC has been confirmed as an upstream regulator of the NOTCH pathway during this process. Inhibiting IL-6, JAK2/STAT3 signaling, or knocking down c-MYC expression attenuated NE phenotypic reprogramming. Collectively, our findings reveal a cascade where fibroblast-secreted IL-6 activates JAK2/STAT3, subsequently triggering c-MYC-mediated NE phenotypic reprogramming via the NOTCH pathway.

To extend the significance of CAFs’ role in NE phenotypic reprogramming, we sought to investigate the immune features in CAF-enriched SCLC. As previous demonstrated, Non-NE SCLC subtypes exhibit greater immune cell infiltration and checkpoint molecule expression, defining an “immune oasis” phenotype^8^. Interestingly, CAF-abundant SCLC with Non-NE characteristics also possess inflamed immune microenvironment. In detail, positive correlations between CAF abundance and specific immune cell infiltration were observed in both cohorts, including T cells, B cells and NK cells, indicated the enhancement of immune surveillance ability. Consistently, the high-CAF infiltration group demonstrated elevated CYT scores, indicating increased CD8+ T cell cytotoxicity^39^. Thus, SCLC with high CAF infiltration seemly presents an “immune hot” profile. Actually, although the immunosuppressive function of CAFs has been prevalently reported, some studies also revealed their immune activation function^40,41^. Additionally, as widely recognized, CAFs were great heterogenous with context-dependent function^16^. Further analyzing functional differences between high and low CAF infiltration groups revealed numerous enriched immune-related pathways in the former. Strikingly, checkpoint molecules like PD-L1 and PD-1 were upregulated in high-CAF infiltration groups. In SCLC, some of immune-related signatures, including APM signature and T-cell-inflamed GEP, have shown promise in predicting survival benefits from ICB therapy in SCLC^42,43^. As we expected, the results shown APM signatures and T-cell-inflamed GEP were elevated in CAF high-infiltration groups, correlating positively with CAF abundance or marker expression. This also suggests that SCLC with high CAF infiltration may possess enhanced antigen presentation capabilities, likely tied to abundant dendritic cells (DCs) shown in Figs. 4 and 7 . Moreover, CAFs also possess capability of antigen presentation as recently reported^22^. Interestingly, the ability of antigen presentation was identified to critical for clinical benefit from ICB therapy in SCLC patients^43^. In all, SCLC with high-infiltration of CAFs has great potential to reach benefit from ICB treatment because of the abundant of cytotoxic immune cells, the up-regulation of checkpoint molecules and the enrichment of immune activation-related signatures. As the limitation, these results derive from LS-SCLC cases, their applicability to ES-SCLC warrants further exploration. Otherwise, CAFs is heterogenous with multiple subtypes, we did not analyze the subtypes of CAFs in SCLC for lacking the indicate data. Given our results, the subtype of apCAF may abundant in CAF-enriched SCLC.

In another side, we investigated the impact of CAFs on drug response in SCLC to explore the underlying significance of CAF-caused phenotypic reprogramming. we predicted SCLC drug sensitivity in the GDSC2 database using George’s cohort’s gene expression profile. A striking discrepancy in drug vulnerability emerged between high and low CAF infiltration groups. displayed heightened resistance to chemotherapy agents like Cisplatin and Irinotecan, consistent with experimental data showcasing fibroblasts’ role in reducing SCLC cell sensitivity to chemotherapy agents. These results were consistent with the notion that SCLC cells convert to Non-NE cells resulted in chemoresistance^6^. In addition, we observed a significant enrichment of the PI3K pathway, previously identified as a promoter of chemoresistance in SCLC^44^, within the CAF-high group (Fig. S5a,b). Furthermore, the expression levels of YAP1 and EPHA2, known to be up-regulated in chemoresistant samples^45,46^, were notably higher in the CAF-high group compared to the CAF-low group. Conversely, the expression of SLFN11, which has been associated with down-regulation in chemoresistant samples^47^, showed significantly lower levels in the CAF-high group compared to the CAF-low group (Fig. S5c,d). These findings provide further confirmation of CAFs’ role in promoting chemotherapy resistance, supported by in vivo data from SCLC. Notably, sensitivity to JAK inhibitors increased with high CAF infiltration, aligning with GSVA and experimental results presenting JAK2/STAT3 pathway activation in CAF-enriched SCLC. Importantly, AZD1480, a JAK inhibitor, effectively reversed CAF-induced SCLC chemoresistance. However, more research is needed to explore JAK inhibitors’ potential in overcoming SCLC chemoresistance. Additionally, promising targeted drugs like PARP inhibitors and AURKA inhibitors exhibited reduced sensitivity in high-CAF infiltration SCLC^48^. For this case, ICB treatment and JAK inhibitor emerged as appealing options for SCLC with abundant CAF infiltration.

In terms of limitations in this study, the role of IL-6 and JAK2/STAT3 pathway during CAF reprograming SCLC phenotype was not confirmed in clinically relevant models or animal models. Actually, CAF-derived IL-6 and JAK/STAT3 pathway have been demonstrated to involve in treatment resistance in multiple other cancers, including pancreatic cancer, cholangiocarcinoma, colorectal cancer and breast cancer^28,49–51^. These studies used clinical samples and mouse models confirmed the role of CAF-derived IL-6 and JAK/STAT3 pathway in treatment resistance. However, similar evidences in SCLC have not been discovered. Further investigations using clinical samples and preclinical models are warranted in the future research.

In conclusion, our comprehensive study unveils a novel mechanism of SCLC cell-CAF crosstalk, highlighting the potential influence of microenvironmental factors on SCLC cell plasticity. Specifically, we have identified fibroblast-derived IL-6 as a mediator of the paracrine regulation responsible for fibroblast-induced NE phenotypic reprogramming in SCLC cells. This reprogramming process is dependent on the JAK2/STAT3/c-MYC pathway. The significance of CAF-induced phenotypic reprogramming from NE to Non-NE is underscored by our findings, as CAF-abundant SCLC displays inflamed immune microenvironment and increased chemoresistance. This study advances our understanding of the heterogeneity within the SCLC microenvironment, offering promising insights into the potential clinical implications of CAFs in SCLC.

## Methods

### Transcriptome data from databases

The RNA-seq data and clinical information of 81 SCLC tumor tissue samples from George and colleagues (U Cologne, Nature 2015)^52^ were downloaded from cBioPortal database (http://www.cbioportal.org) according to the guidance. Dataset of gene expression from SCLC tumor tissue (GSE60052) was downloaded from Gene Expression Omnibus (GEO, https://www.ncbi.nlm.nih.gov/geoprofiles/).

### Software

R Studio (version 4.3.2) were used for bioinformatics analyses. R packages used for estimating of microenvironmental cell fractions in this study were xCell (version 1.1.0)^53^, MCP-counter (version 1.2.0)^54^ and EPIC (version 1.1.7)^55^. Immune cells were estimated by CIBERSORTx (https://cibersortx.stanford.edu/)^56^. ESTIMATE algorithm (version 1.0.13) was used to calculate the immune score^57^. ComplexHeatmap package (version 2.15.4) was used to cluster the CAF infiltration^58^. Gene Set Variation Analysis (GSVA) was performed using GSVA package (version 1.48.3) in RNA-seq data^59^, Hallmark gene sets were obtained from the MSigDB Collections (http://www.gsea-msigdb.org/gsea)^60^. GSVA results were compared between high- and low-CAF infiltration group using limma package (version 3.56.2). Gene Set Enrichment Analysis (GSEA) for NE and Non-NE gene lists in RNA-seq data between high- and low-CAF infiltration group was preformed using GSEA software (version 4.0.2)^61^, the NE and Non-NE gene lists were obtained from Zhang’s study^62^. The drug sensitivity scores which were used to indicate the half-maximal inhibitory concentration (IC50) of all drugs in the Genomics of Drug Sensitivity in Cancer v2 (GDSC2) database (https://www.cancerrxgene.org/, accessed on 7 February 2023) were predicted using the R package “oncoPredict” (version 0.2) based on RNA-seq data from George’ cohort^63,64^. The lower sensitivity score indicated the higher drug sensitivity. Sensitivity score compared between high- and low-CAF infiltration group using limma package. All packages used in this study was obtained from GitHub (https://github.com/).

### SCLC subtyping

Molecular subtypes of SCLC-A, SCLC-N, SCLC-P and SCLC-Y subtypes were identified using the single highest gene expression of ASCL1, NEUROD1, POU2F3 and YAP1 as previous reported^7^, the heatmap of expression of these four factors was made by ComplexHeatmap.

### CAF clustering

Classic CAF markers (PDGFRA, PDGFRB, PDPN, FAP, THY1, COL1A1, COL1A2, COL3A1, ACTA2, S100A4) from previous studies^34,65,66^ and CAF abundance estimated by xCell (Fibroblast_X), MCP-counter (Fibroblast_M) and EPIC (Fibroblast_E) were adopted for CAF clustering in George’ cohort and GSE60052 dataset using the ComplexHeatmap package. Cases of these two datasets were respectively divided into high- and low- CAF infiltration groups.

### Tissue arrays and immunohistochemical (IHC) staining

Tissue arrays (R801001) containing 80 tissue samples from 37 SCLC patients and 3 healthy controls were purchased from Zhongkeguanghua bioaitech company (Xian Shanxi, China). The detailed information about this tissue array was listed in Supplementary Table S1.

Ultra-sensitive SP IHC Kit (MXB biotechnologies, Fuzhou, Fujian, China) was used for IHC staining to measure the expression of α-SMA, CD56 and REST. Briefly, tissue array slides were rehydrated and blocked peroxidase activity with 3% H_2_O_2_ for 20 min.

Then slides were boiled for 5 min in citrate buffer (pH 6.0) to retrieval antigen. The next steps were performed following the manufacture’s instruction of IHC kit. Primary antibodies used in this experiment were α-SMA (Abclonal, 1:50, Wuhan, Hubei, China), CD56 (Proteintech, 1:6000, Chicago, IL, USA) and REST (Proteintech, 1:400, Chicago, IL, USA). IHC results were evaluated as previous reported^67^, by assessing (a) the proportion of positively cells per the whole cells (0, < 5%; 1, 6–25%; 2, 26–50%; 3, 51–75%; 4, 76–100%) and (b) the intensity of staining (0, negative; 1, weak staining; 2, medium staining; 3, strong staining). The IHC score was calculated by a × b.

### Cell culture and treatments

Human SCLC cell lines (H69, H2227, H1092, H446, 21H, H196 and SBC-5) were obtained from National collection of Authenticated cell cultures (Shanghai, China) or Procell Life Science and Technology company (Wuhan, Hubei, China) in 2021. HEK-293T cell line was a gift from Prof. Jie Ma (College of Pharmacy, Jilin university). Cells were cultured in Dulbecco’s Modified Eagle Medium (Sigma Aldrich, St. Louis, MO, USA) or RPMI-1640 medium (Sigma Aldrich, St. Louis, MO, USA) which were supplemented with 10% (*v/v)* FBS (Vivacell, Shanghai, China) and 100 IU/mL of penicillin and streptomycin (Biological Industries, Kibbutz Beit Haemek, Israel). Human lung fibroblasts (MRC-5, HFL1) were purchased from Zhong Qiao Xin Zhou Biotechnology company (Shanghai, China) and Procell Life Science and Technology company respectively in 2022 and cultured with expansion medium. Fibroblasts used in all experiments were within 10 passages. Cells were maintained at 37 °C in a 5% CO_2_ in air humidified incubator. STR identification for all cells were correct before we buy.

For co-culture assay, SCLC cells and fibroblasts were respectively seeded in 6-well plates and in polyester membrane of transwell insert with 0.4-μm pore size with 1:1 ratio. SCLC cells cultured separately were set as controls.

Other details of cell treatments were described in Supplementary information.

### Cytokine antibody arrays

Serum-free media from separately cultured MRC-5, H2227 and co-cultured MRC-5/H2227 cells were collected after cultured for 48 h, Human Cytokine Antibody Array C5 (Raybiotech, Peachtree Corners, GA, USA) were used according to the manufacturer’s instruction. In brief, the collected media was incubated with blocked membranes overnight at 4℃. After washing, the membranes were incubated with biotinylated antibody for 2 h at room temperature, washing again and HRP-Streptavidin incubation was performed for 2 h at room temperature. Chemiluminescence was detected by GeneGnome XRQ NPC imaging system (Syngene, Cambridge, UK) and gray values of dots were analyzed by Image J software.

### Dual-luciferase reporter gene assay

The dual-luciferase reporter assay was performed as previous described^68^. Briefly, the 5′-flanking region (− 900 to  + 100) of MYC was cloned into the luciferase reporter gene vector pGL4.20 (MiaolingBio, Wuhan, Hubei, China) and confirmed by DNA sequencing. HEK-293T cells were transfected with EF.STAT3C.Ubc.GFP plasmid (Addgene, Watertown, MA, USA) to constitutively activate STAT3^69^ followed by co-transfection with pGL4.20 with or without MYC promoter fragment and the renilla vector pGL4.74 in a ratio of 1:50. Luciferase activities were determined by Dual Luciferase Reporter Gene Assay Kit (YEASEN, Shanghai, China) and detected with CLARIOstar microplate reader. The renilla luciferase activity served as a reference.

### Chromatin immunoprecipitation (ChIP)-qPCR

The ChIP assay was performed using ChIP Assay Kit (Beyotime, Beijing, China) according to the manufacturer’s instruction. In brief, H69 and H2227 cells were separately cultured or co-cultured for 6 days, then cells were cross-linked with 1% fresh formaldehyde and 125 mM glycine was added to stop linking. Cells were harvested and sonicated in lysis buffer by ultrasonic cell disruptor. DNA fragments were immunoprecipitated with STAT3 (Proteintech, Chicago IL, USA) and IgG Beyotime, Beijing, China) antibodies. 5 M NaCl was used to reverse cross-links, RNase A and Protein K were used to digest RNA and protein. Finally, DNA was purified using DNA Purification Kit (Beyotime, Beijing, China) and used for qRT-PCR analysis. Primers were listed in Supplementary Table S2.

### Scores and Signatures

#### NE score

NE score was calculated according to the way as previous reported^62^. Briefly, Pearson correlations between expression of the 50 genes from the NE and Non-NE gene lists in the George’ cohort or in the GSE60052 dataset with the genes in the NE (or Non-NE) expression vectors from Zhang’s study were analyzed and NE score for every cases was calculated by the formula: NE score = (correl NE − correl Non-NE)/2. NE score > 0 indicated as NE phenotype and NE score < 0 indicated as Non-NE phenotype.

#### Cytolytic activity (CYT) score

The CYT score was calculated as the geometric mean of granzyme (GZMA) and perforin (PRF1) expression^39^.

#### Antigen presentation machinery (APM) signature

The score of APM signature was calculated as the median value of Z-scored expression of APM signature genes in George’ cohort or GSE60052 dataset^43^.

#### T-cell-inflamed gene expression profile (GEP)

The score of T-cell-inflamed GEP was calculated as weighed sum of the normalized 18 genes expression values by log2(count + 1)^42^, the weightings for each gene were obtained from a published patent (WO2016094377, https://patentscope.wipo.int/search/en/detail.jsf?docId=WO2016094377&tab=PCTCLAIMS).

Details for gene signatures are shown in Supplementary Table S3.

#### Ethics declarations

The tissue arrays were obtained from ZK bioaitech company, informed patient consent was obtained prior to collection. The study was approved by Ethics Committee of Changsha Yaxiang Biotechnology Co., Ltd (Csyayj20231120). All methods were performed as per the relevant regulations and the Declaration of Helsinki.

### Statistical analysis

Statistical analysis in this study was performed using R studio and GraphPad Prism (version 8.2.1). Significance of data were determined by two-tailed Student’s *t*-test or Mann–Whitney *U* test or ANOVA test. Pearson’s correlation was applied to assess the correlation between indicated factors. Data presented as mean ± SEM, *P* values < 0.05 considered statistically significant.

### Supplementary Information

Supplementary Table S1.

Supplementary Information.

## Data Availability

All data discussed in this manuscript are included in the main manuscript text or supplementary materials.
