# Supplementary material for: Dynamic phenotypic reprogramming and chemoresistance induced by lung fibroblasts in small cell lung cancer
Source: Sci Rep. 2024 Feb 5;14:2884. doi: 10.1038/s41598-024-52687-z (PMC10838940; doi:10.1038/s41598-024-52687-z)

Title: Dynamic phenotypic reprogramming and chemoresistance induced by lung fibroblasts in small cell lung cancer

Yuanhua Lu [1], Hui Li [2], Peiyan Zhao[2], Lin Tian[1], Yan Liu[2], XiaoDan Sun[3], Ying Cheng[2,4]*

[1]Postdoctoral Research Workstation, Jilin Cancer Hospital, Changchun, China;

[2]Medical Oncology Translational Research Lab, Jilin Cancer Hospital, Changchun, China;

[3] Department of 1st Gynecologic Oncology Surgery, Jilin Cancer Hospital, Changchun, China;

[4] Department of Medical Thoracic Oncology, Jilin Cancer Hospital, Changchun, China.

*Correspondence: No. 1066, Jinhu Road, High-tech District, Changchun City, Jilin Province, China; Post code: 130012; E-mail: chengying@csco.org.cn; Tel.: +86-431-80596315.

1 Supplementary materials and methods

1.1 Cell treatments

For JAK2/STAT3 inhibitor treatment, H69 and H2227 were pre-treated with 0.8 μM AZD-1480 (MedChemExpress, NJ, USA) for 24 h, then co-cultured with fibroblasts or separately cultured with complete medium containing 0.8 μM AZD-1480, then expression of NE genes or c-MYC, HES1 or cell viability were determined.

1.2 Small interfering RNA (siRNA) transfection

MRC-5 or HFL1 were seeded in transwell inserts (Labselect, Hefei, Anhui, China) which put into 6 or 24-well plate for 24 h, then 80 nM (6 well plate) or 20 nM (24 well plate) siRNA (GenePharma, Suzhou, Jiangsu, China) diluted with Opti-MEM medium (Gibco, Carlsbad, CA, USA) was transfected to MRC-5 or HFL1 cells by Lipofectamine RNAiMAX reagent (Invitrogen, Carlsbad, CA, USA) for 6 h. Another 24 h later, MRC-5 or HFL1 transfected with siRNA were co-cultured with H69 or H2227 cells for another 6 days. Sequences of siRNA targeted to IL6, IGFBP1, CCL20 and negative control were listed in Supplementary Table S4.

1.3 Lentivirus packaging and infection

Well grown HEK-293T cells were seeded in 10-cm dish for 24 h before plasmids transfection, then 10 μg expression plasmid GV248 carried MYC RNAi fragment or Scramble fragment (Genechem, Shanghai, China) and 10 μg lentivirus packaging plasmids (7.5 μg psPAX+ 2.5 μg pMD2G) (Fenghui Biotechnology, Changsha, Hunan, China) were diluted with opti-MEM medium and transfected in HEK-293T cells by Lipofectamine 2000 reagent (Invitrogen, Carlsbad, CA, USA) for 6 h. Transfected cell cultured for another 48 h, then supernatant was collected and centrifuged to obtain the lentivirus. Virus was subpackaged and stored at − 80 ° C for specified experiments. The shRNA sequence targeted to *MYC* was listed in Supplementary Table S4.

1.4 Cell proliferation analysis

SCLC cell line H69 and H2227 which been treated with AZD-1480 or lentivirus or not were seeded in 24-well plates at 2×10^4 cells /well and co-cultured with MRC-5 or HFL1 which were untreated or transfected with siRNA, or cultured separately. 5 days later, cell viability was determined by Cell Counting Kit-8 (CCK-8) reagent (Beyotime, Beijing, China), the optical absorbance was measured at 450 nm by CLARIOstar microplate reader (BMG Labtech, Offenburg, Germany).

For clone formation test, SCLC cell line H2227 were seeded in 24-well plated at 500 cells/well and co-cultured with MRC-5 and HFL1 respectively or separately cultured for 7 days. The cell clones were washed by PBS and fixed by 4% paraformaldehyde, and then stained by 0.1 % (m/v) crystal violet for 15 min. Cell clones of every well were photographed and counted by Image J software.

1.5 Cell viability assay

SCLC cells co-cultured with fibroblasts for 7 days and then seeded in 96- well plates and exposed to Cisplatin or Etoposide for another 48 h. Cell viability was measured by CCK-8 reagent. The inhibition rate was calculated with the formula as: inhibition rate=1-OD450 (drug treatment group)/OD450 (control group) ×100%.

1.6 Western blot analysis

To extract the total protein of cells, RIPA reagent supplemented with protease inhibitor and phosphatase inhibitors (Beyotime, Beijing, China) was added to SCLC cells and lysed on ice for 30 min. The concentrations of supernatant were determined using BCA protein assay kit (Beyotime, Beijing, China). The same amount of protein was separated by SDS-PAGE and transferred onto PVDF membranes (Millipore, Darmstadt, Germany). The blots were blocked and cut prior to hybridisation with antibodies overnight at 4 ℃. The blots incubated with HRP-conjugated secondary antibodies and then chemiluminescence of blots were stimulated by ECL reagent (Beyotime, Beijing, China) and detected by GeneGnome XRQ NPC imaging system. Quantitative analysis of blots was performed using Image J software. The details of antibodies were listed in Supplementary Table S5.

1.7 RNA extraction and quantitative real-time PCR (qRT-PCR)

Total RNA was extracted using TRIzol reagent (Invitrogen, Carlsbad, CA, USA). ReverTra Ace qPCR RT Master Mix (TOBOYO, Osaka, Japan) was used to reverse transcribe 1 μg RNA to cDNA. Then cDNA was used to perform qRT-PCR with SYBR Green qPCR Mix (Beyotime, Beijing, China) in an Agilent Mx3000P qPCR System (Palo Alto, CA, USA). The relative mRNA expressions were calculated using the 2–ΔΔCt method. GAPDH was used as a loading control. Primers were generated by Beijing Dingguo Changsheng Biotechnology (Beijing, china) and sequences were listed in Supplementary Table S2.

2 Supplementary Figures and Tables

2.1 Supplementary Tables

Supplementary Table S2: Sequences of primers for qRT-PCR and ChIP-PCR

Genes	Sequences
GAPDH	F: 5’-GCCGCACCTCAGCTTATTATG-3’
	R: 5’-AAGTGGTCGTTGAGGGCAATG-3’
IL6	F: 5’-ACTCACCTCTTCAGAACGAATTG-3’
	R: 5’-CCATCTTTGGAAGGTTCAGGTTG-3’
CCL20	F: 5’-GCGAATCAGAAGCAGCAAGCAA-3’
	R: 5’-TGTTTTGGATTTGCGCACACAG-3’
Genes	Sequences

IGFBP1	F: 5’-TTGGGACGCCATCAGTACCTA-3’<br>R: 5’-TTGGCTAAACTCTCTACGACTCT-3’
ASCL1	F: 5’-CGCGGCCAACAAGAAGATG-3’<br>R: 5’-CGACGAGTAGGATGAGACCG-3’
SYP	F: 5’-TTAGTTGGGGACTACTCCTCG-3’<br>R: 5’-GGCCCTTTGTTATTCTCTCGGTA-3’
NCAM1	F: 5’-GGCATTTACAAGTGTGTGGTTAC-3’<br>R: 5’-TTGGCGCATTCTTGAACATGA-3’
IL6R	F: 5’- CATGTGCGTCGCCAGTAGT-3’<br>R: 5’- AGCTCAAACCGTAGTCTGTAGA-3’
MYC (ChIP)	F: 5’-CACAAGGGTCTCTGCTGACTC-3’<br>R: 5’-TCAAAGGTGCTAGACGGGAG-3’

Supplementary Table S3: Gene signatures

Signatures	Genes
NE genes	BEX1, ASCL1, INSM1, CHGA, TAGLN3, KIF5C, CRMP1, SCG3, SYT4, RTN1, MYT1, SYP, KIF1A, TMSB15A, SYN1, SYT11, RUNDC3A, TFF3, CHGB, FAM57B, SH3GL2, BSN, SEZ6, TMSB15B, CELF3.
Non-NE genes	RAB27B, TGFBR2, SLC16A5, S100A10, ITGB4, YAP1, LGALS3, EPHA2, S100A16, PLAU, ABCC3, ARHGDIB, CYR61, PTGES, CCND1, IFITM2, IFITM3, AHNAK, CAV2, TACSTD2, TGFBI, EMP1, CAV1, ANXA1, MYOF.
Signatures	Genes

Cytolytic activity (CYT)	GZMA, PRF1.
Antigen presentation machinery (APM) signature	HLA-A, HLA-B, HLA-C, B2M, TAP1, TAP2.
T-cell-inflamed gene expression profile (GEP)	PSMB10, HLA-DQA1, HLA-DRB1, CMKLR1, HLA-E, NKG7, CD8A, CCL5, CXCL9, CD27, CXCR6, IDO1, STAT1, TIGIT, LAG3, CD274, PDCD1LG2, CD276.

Supplementary Table S4: Sequences of siRNAs and shRNA.

Targets	Sequences
siIL6-1	5’-CUGUGCAGAUGAGUACAAATT-3’
siIL6-1	5’-GACCCAACCACAAAUGCCATT-3’
siCCL20	5’-CCGUAUUCUUCAUCCUAAATT-3’
siIGFBP1	5’-CCAGAGAGCACGGAGAUAATT-3’
Negative control	5’-UUCUCCGAACGUGUCACGUTT-3’
shMYC	5’-CCCAAGGTAGTTATCCTTAAA-3’
Scramble	5’- TTCTCCGAACGTGTCACGT -3’

Supplementary Table S5: Information of antibodies

Antibodies	Sources
GAPDH	Wanleibio (Shengyang, Liaoning, china)
β-actin	Wanleibio (Shengyang, Liaoning, china)
Antibodies	Sources

ASCL1	CST (Danvers, Mass, USA)
NEUROD1	CST (Danvers, Mass, USA)
c-MYC	CST (Danvers, Mass, USA)
HES1	CST (Danvers, Mass, USA)
SYP	CST (Danvers, Mass, USA)
pSTAT3 (Ser727)	CST (Danvers, Mass, USA)
STAT3	CST (Danvers, Mass, USA)
pJAK2 (Tyr1007)	CST (Danvers, Mass, USA)
JAK2	CST (Danvers, Mass, USA)
REST	Santa Cruz (Santa Cruz, CA, USA)
INSM1	Abclonal (Wuhan, Hubei, China)
HRP-conjugated goat anti-Rabbit IgG (H+L)	Wanleibio (Shengyang, Liaoning, china)
HRP-conjugated goat anti-Mouse IgG (H+L)	Beyotime (Beijing, China)

2.2 Supplementary Figures

Supplementary Figure S1

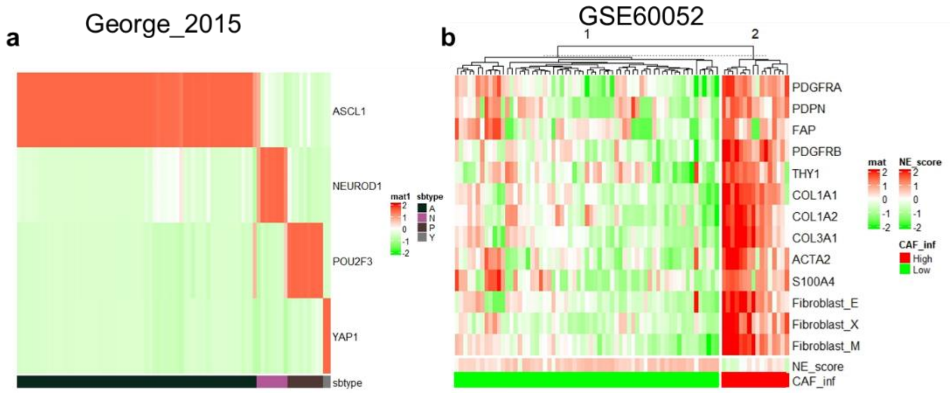

Subtyping SCLC cases and clustering CAF infiltration. (a) Heatmap displayed expression of ASCL1, NEUROD1, POU2F3 and YAP1 in George's cohort (n=81); (b) The expression of CAF markers and abundance of CAFs and the clustering results of GSE60052 dataset (n=79) were displayed in heatmap, NE scores were shown under the heatmap.

Supplementary Figure S2

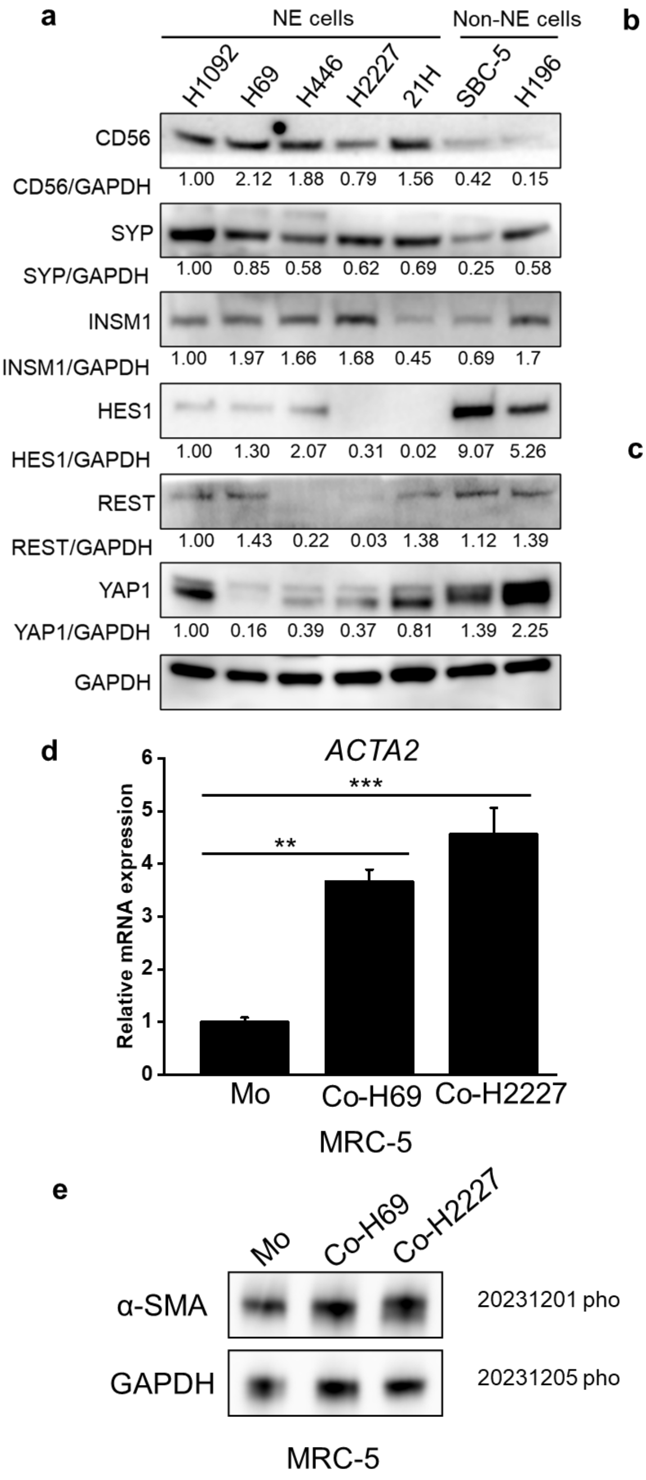

Identification of NE and Non-NE cells and evaluation the effect of fibroblasts on proliferation of SCLC cells. (a) Western blot evaluated the expression of NE markers (CD56, SYP, INSM1) and Non-NE markers (HES1, REST, YAP1) in SCLC cells, the blots were cropped prior to hybridisation with antibodies, original images were displayed in Supplementary information; (b) Cell proliferation was

determined by CCK-8 reagent in H69 cells which separately cultured or co-cultured with fibroblasts (n=3); (c) Colony formation assay was performed to evaluate the proliferation of H2227 cells upon co-culture with fibroblasts or cultured individually (n=3). (d-e) The mRNA expression of ACTA2 determined by qRT-PCR (n=3) and protein expression of α -SMA evaluated by western blot of mono- and co-cultured MRC-5 cells. The blots displayed in (e) were cropped prior to hybridisation with antibodies, original images were displayed in Supplementary information. * $P < 0.05$, *** $P < 0.001$.

Supplementary Figure S3

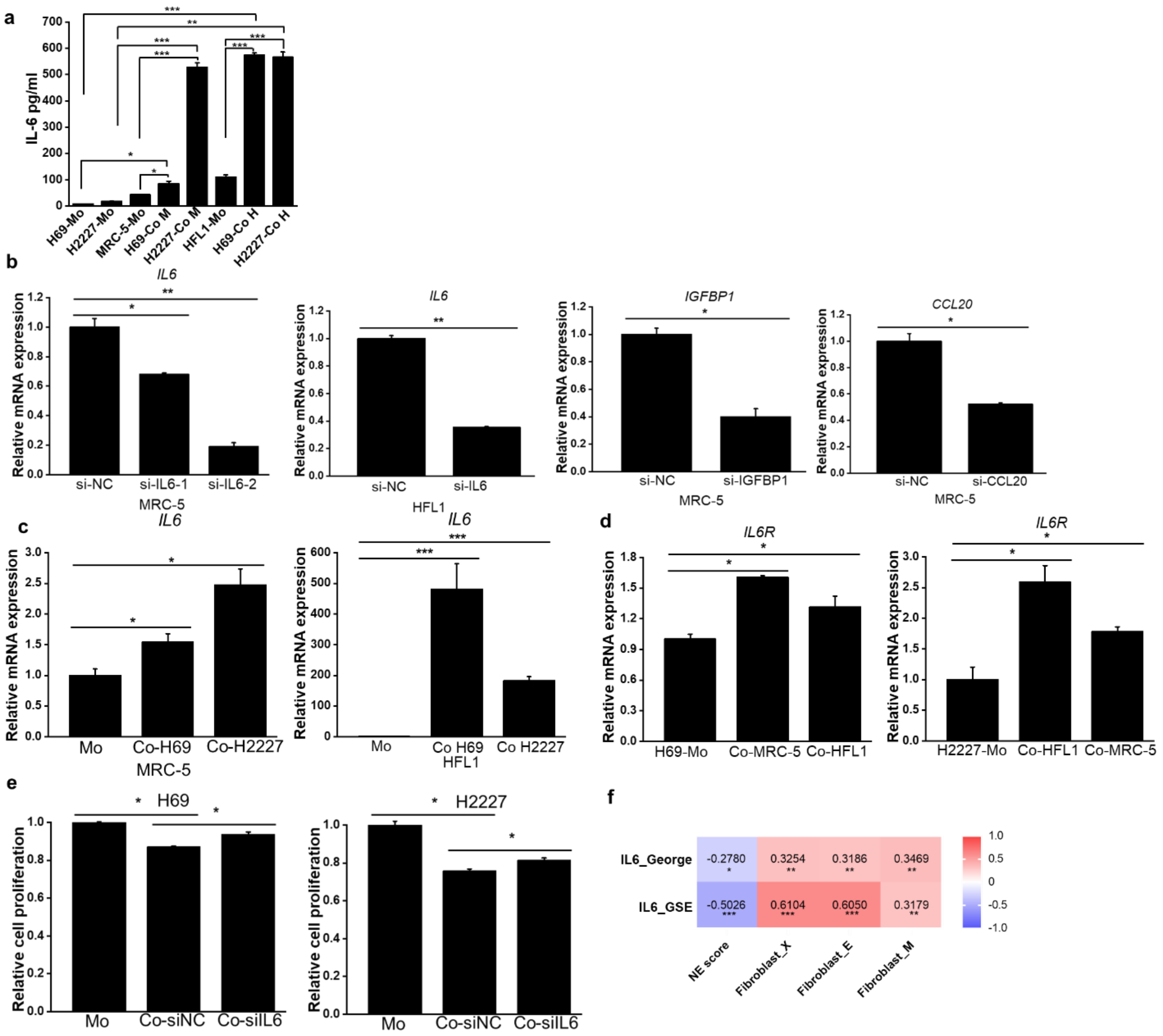

Exploration of IL-6 and IL-6R in CAF-induced NE phenotypic reprogramming. (a) Secreted IL-6 from separately cultured MRC-5, HFL1, H69 and H2227 cells or co-cultured cells were determined by ELISA (n=4); (b) Expression of IL6, IGFBP1 and CCL20 was determined by qRT-PCR in MRC-5 and HFL1 cells upon transfection with indicated siRNA (n=3). (c, d) Expression of IL6 (c) and IL6R (d) was tested by qRT-PCR in MRC-5 and HFL1 cells or H69 and H2227 cells respectively upon co-culture with fibroblasts or cultured individually (n=3); (e) Cell proliferation was determined by CCK-

8 assay after transfection with siRNA tarted to IL6 or negative control (NC); (f) Pearson’s correlation of expression of IL6 with NE score and CAF abundance in George’s cohort and GSE60052 dataset. * $P < 0.05$, ** $P < 0.01$.

Supplementary Figure S4

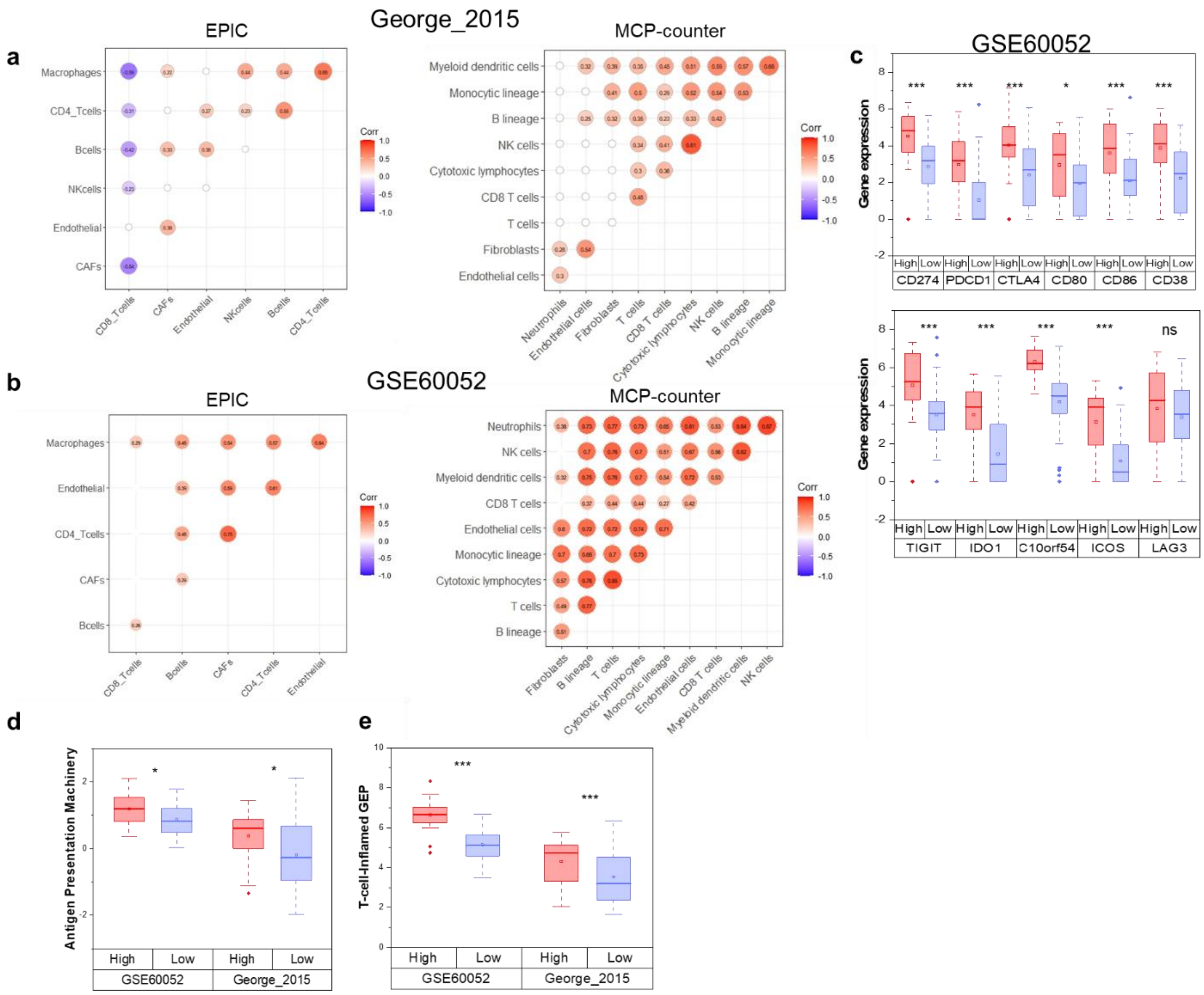

Association of CAFs with immune features in SCLC cohorts. (a, b) Pearson’s correlation of CAF abundance with immune cell abundance in George’s cohort (a) and GSE60052 dataset (b); (c) Expression of checkpoint molecules of high- and low-infiltration groups in GSE60052 dataset; (D-E) APM signature (d) and T-cell-inflamed GEP (e) of high- and low-infiltration groups in George’s cohort and GSE60052 dataset. * $P < 0.05$, ** $P < 0.01$, *** $P < 0.001$.

Supplementary Figure S5

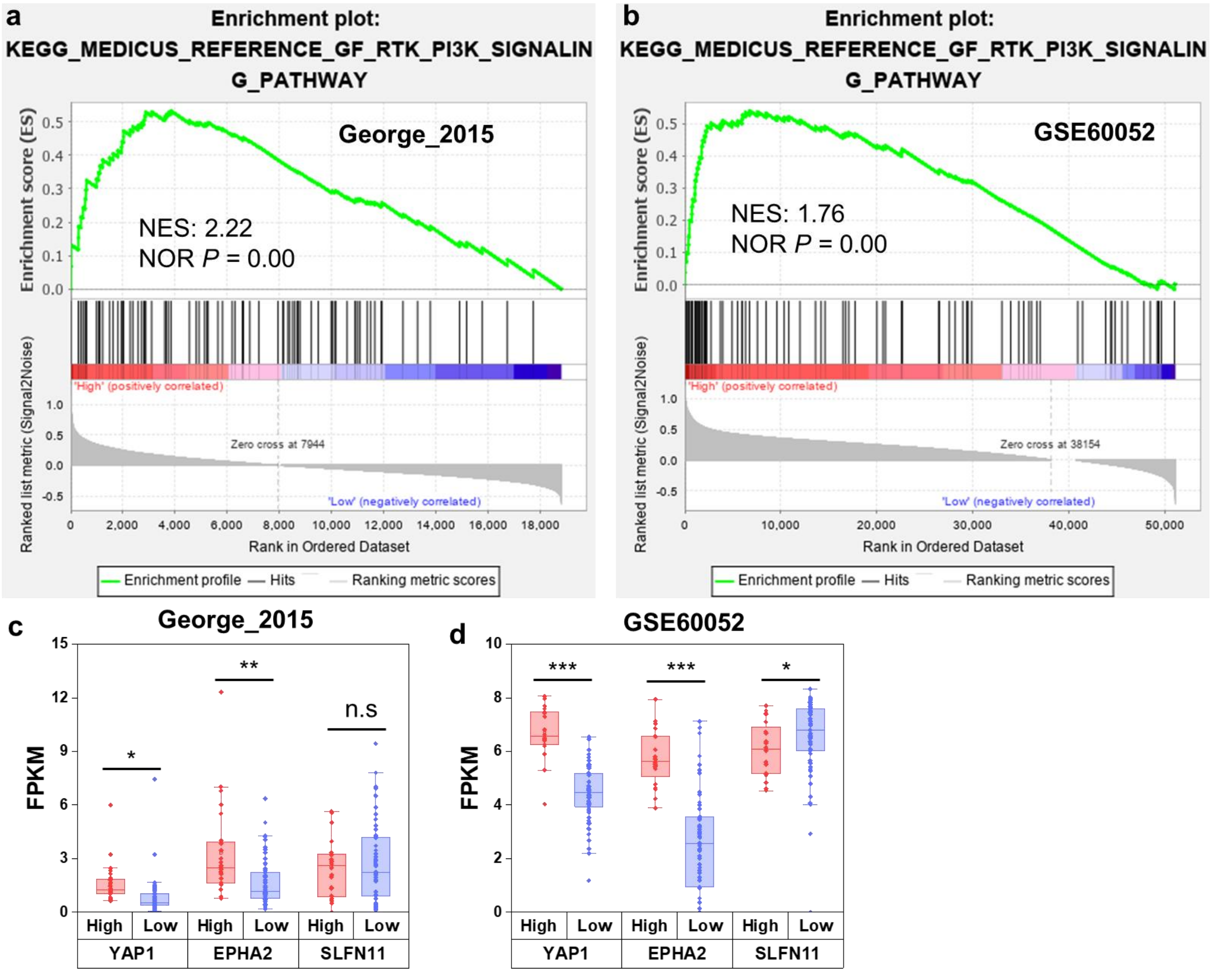

Chemoresistance related pathway and genes in SCLC cohorts. (a-b) GSEA with normalized enrichment score (NES) and nominal *P*-values for KEGG pathway in CAF high- and low-infiltration group in George's cohort (a) and GSE60052 dataset (b); (c-d) Expression of Chemoresistance related genes of CAF high- and low-infiltration groups in George's cohort and GSE60052 dataset. * $P < 0.05$, ** $P < 0.01$, *** $P < 0.001$.

Original images

Figure 2b

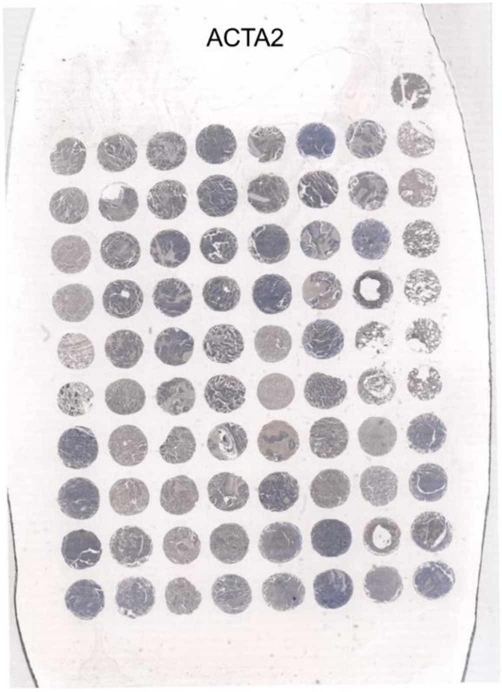

Figure 2b

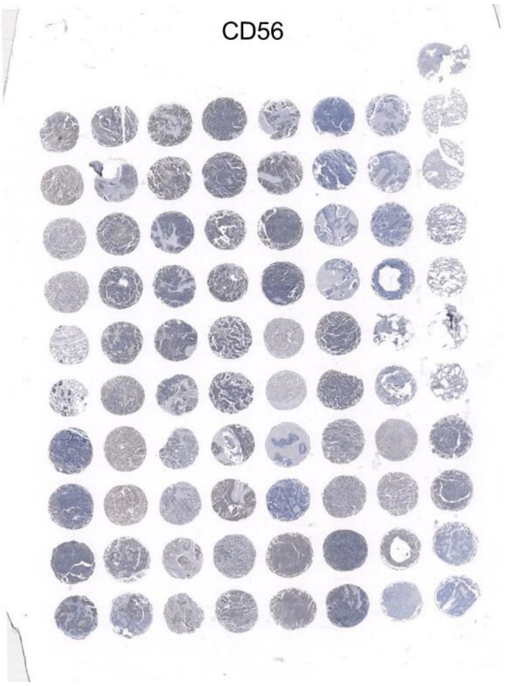

Figure 2b

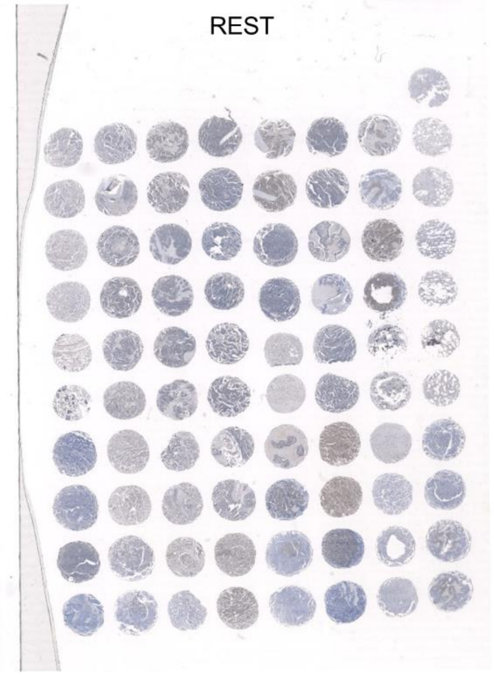

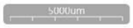

Figure 3c

Low contrast

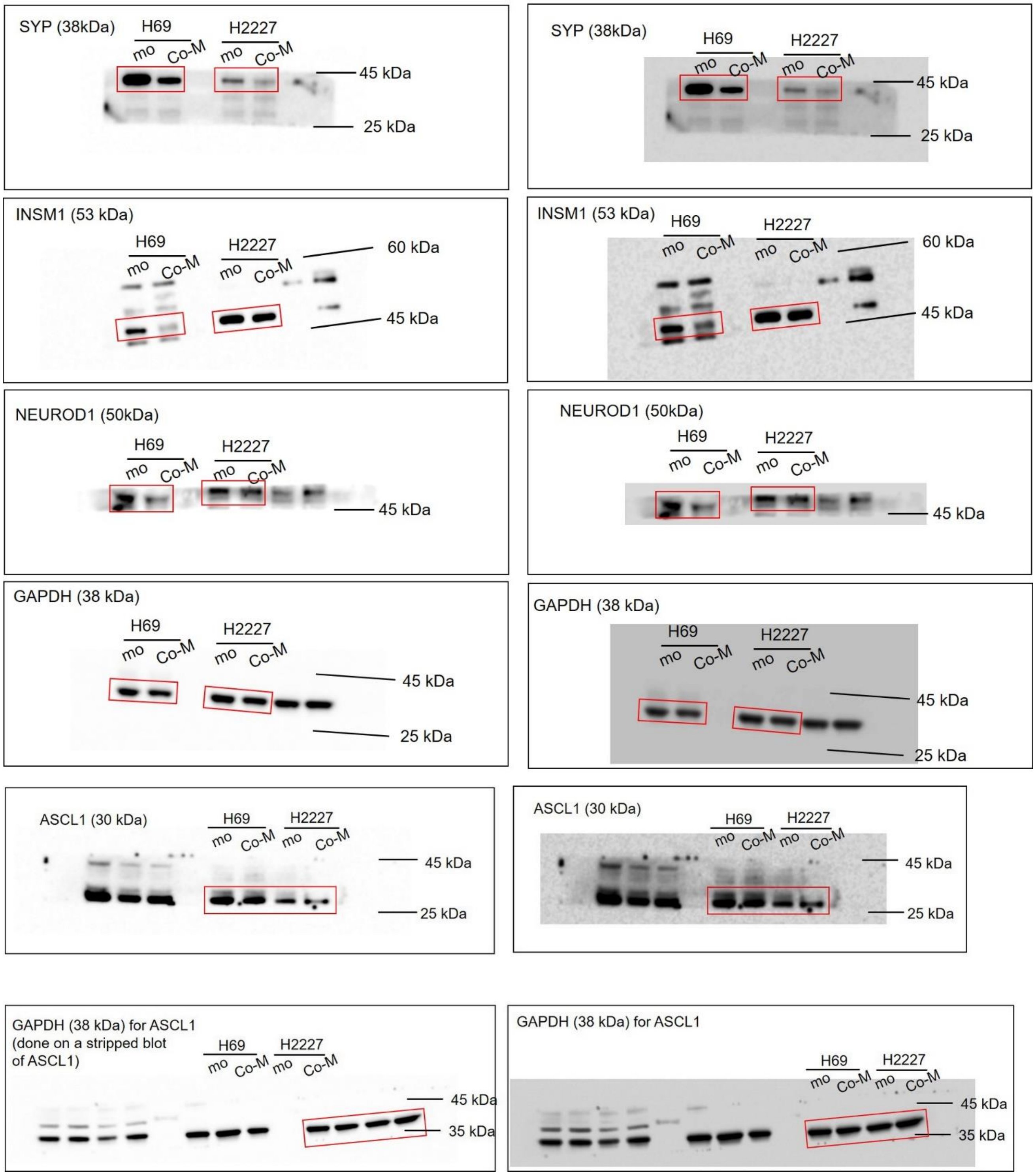

Figure 3c

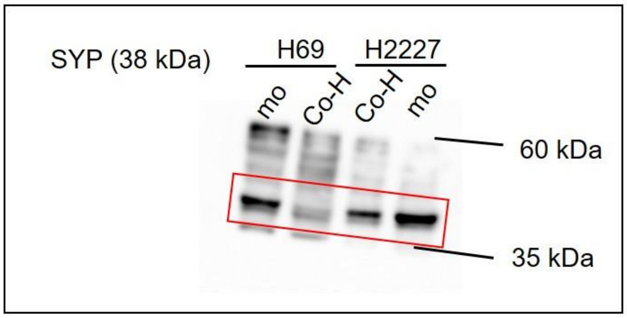

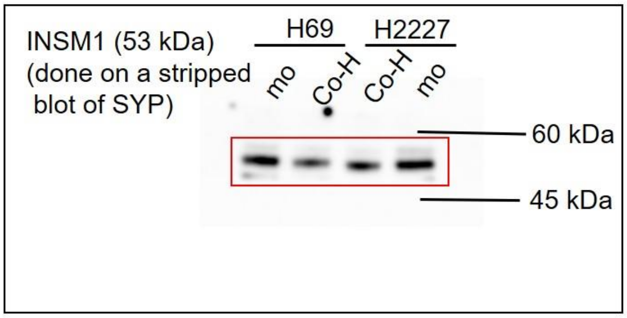

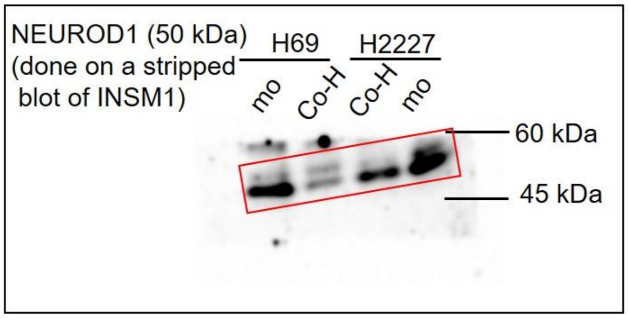

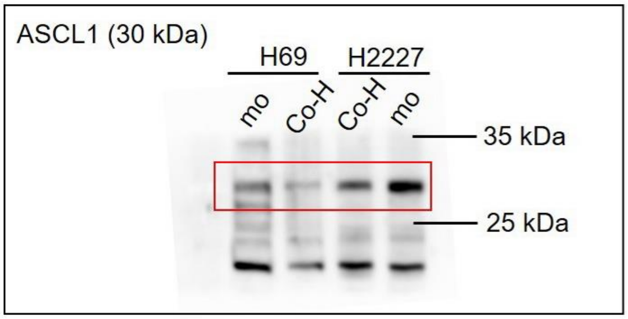

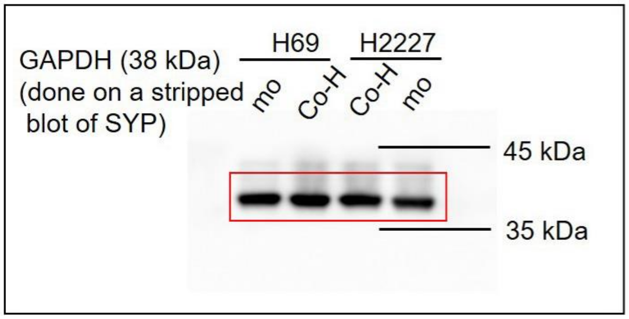

Low contrast

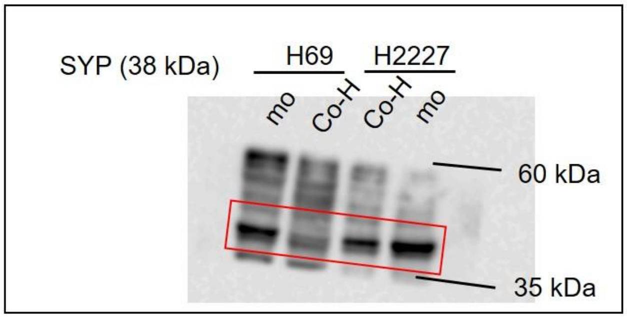

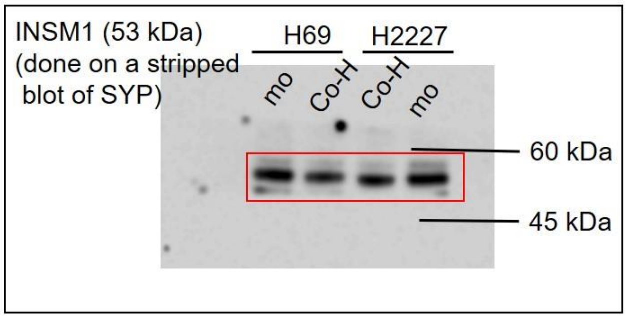

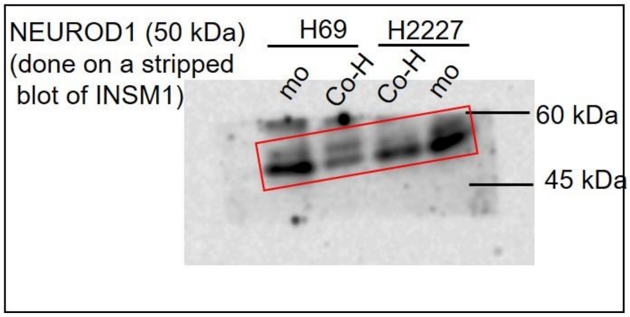

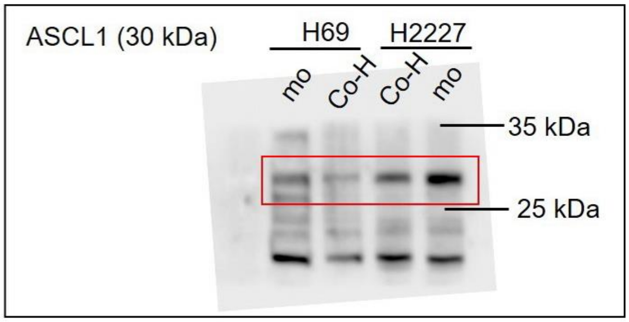

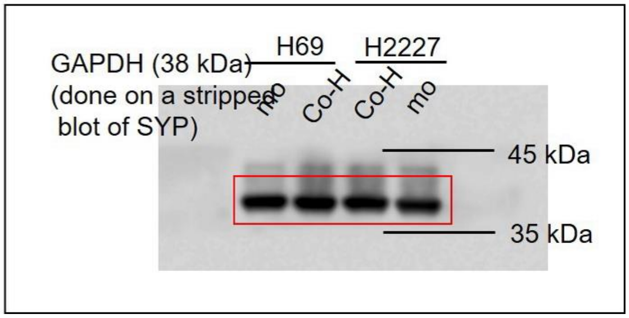

Figure 3g

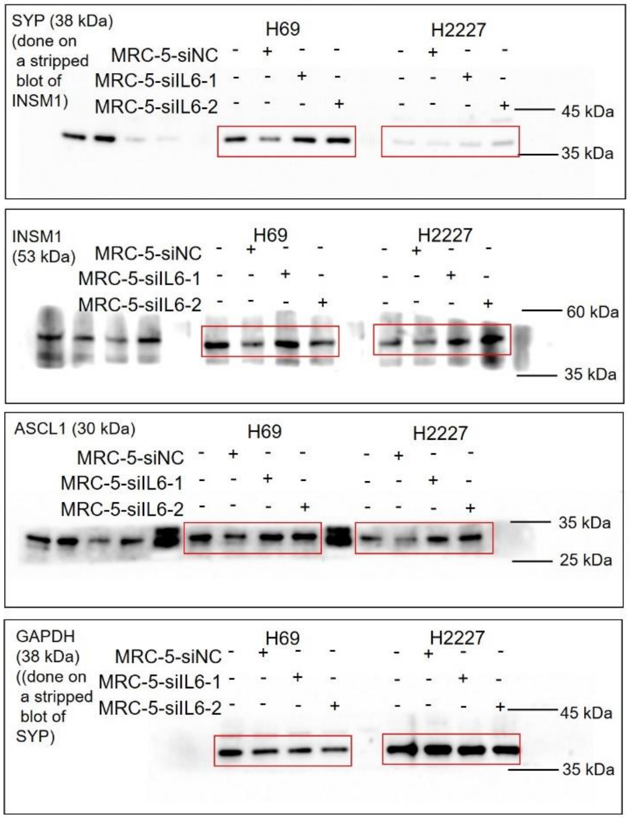

Low contrast

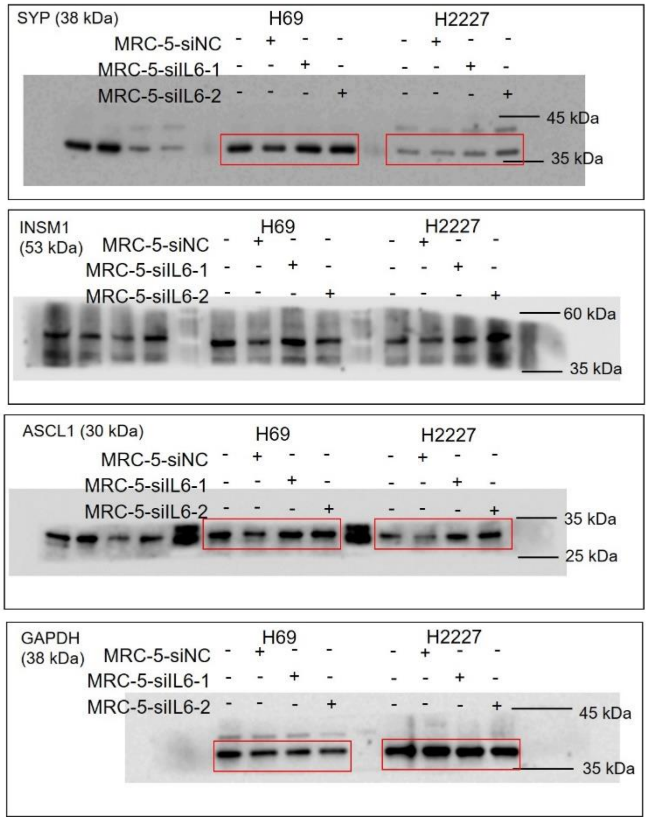

Figure 4c

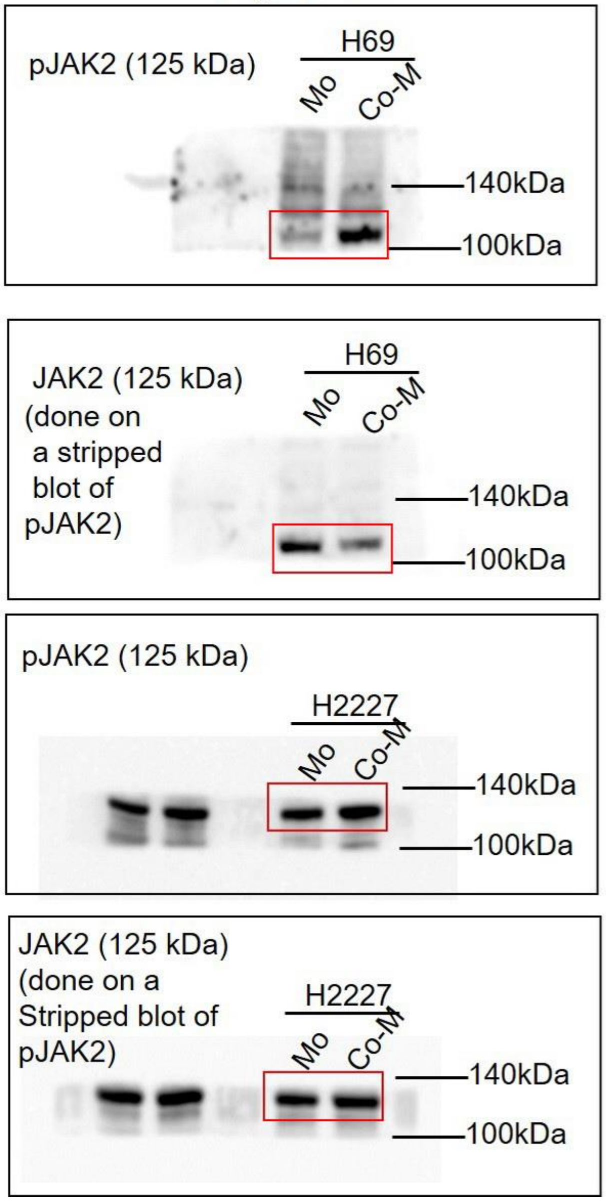

Low contrast

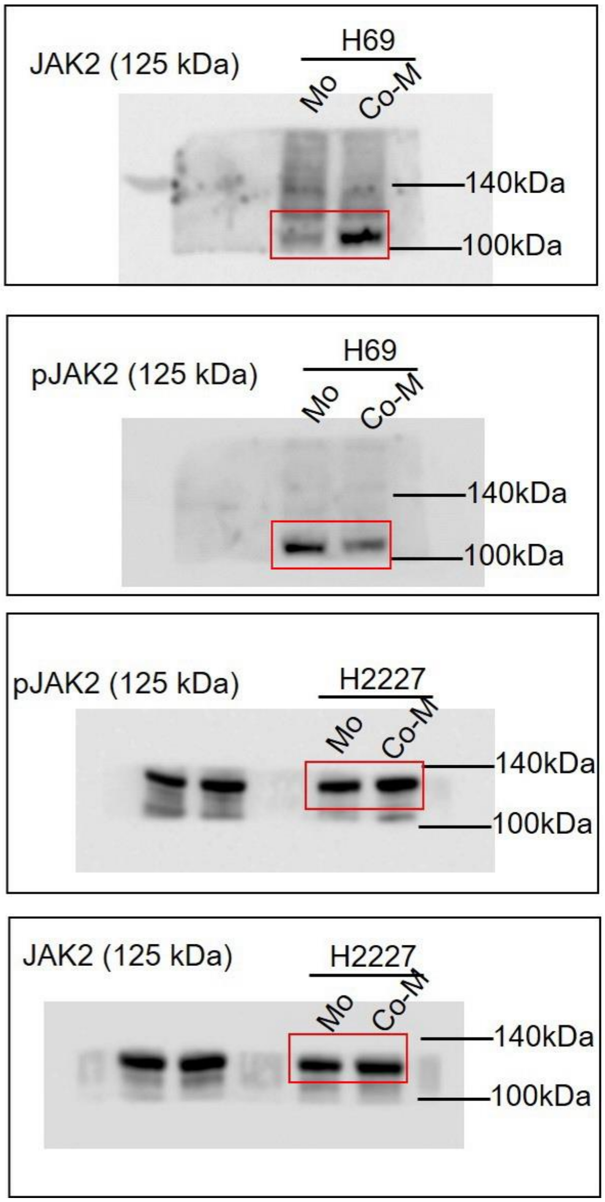

Figure 4c

Low contrast

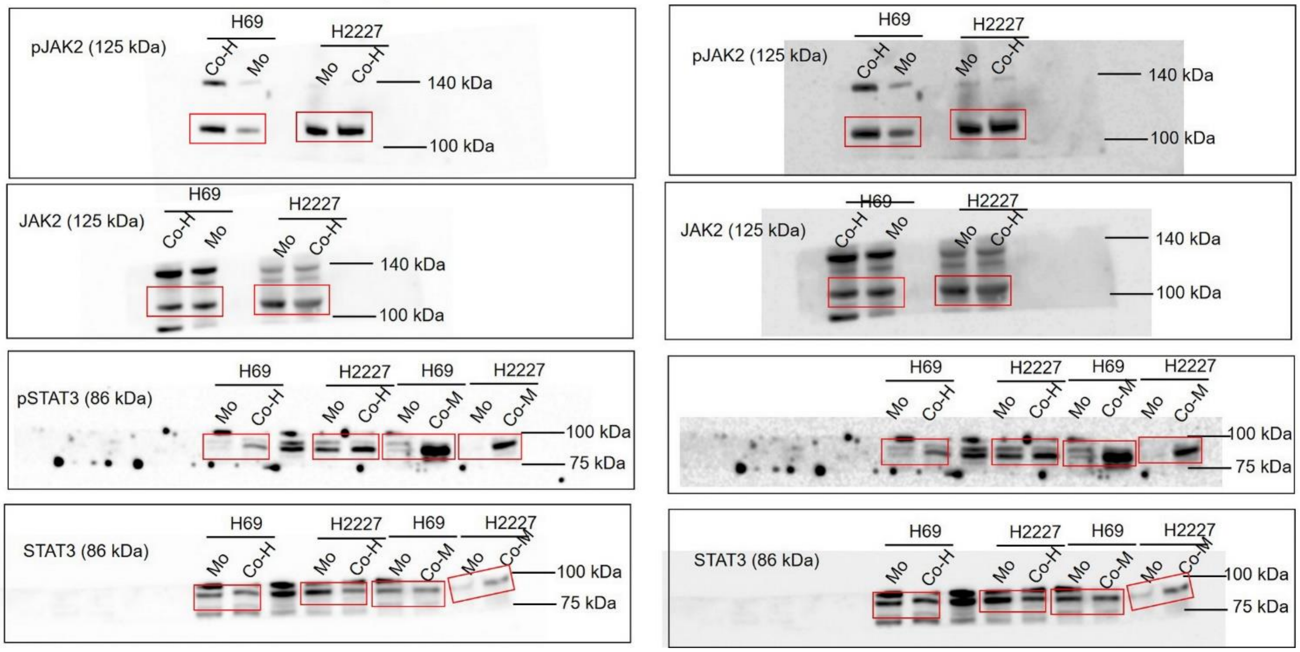

Figure 4d

Low contrast

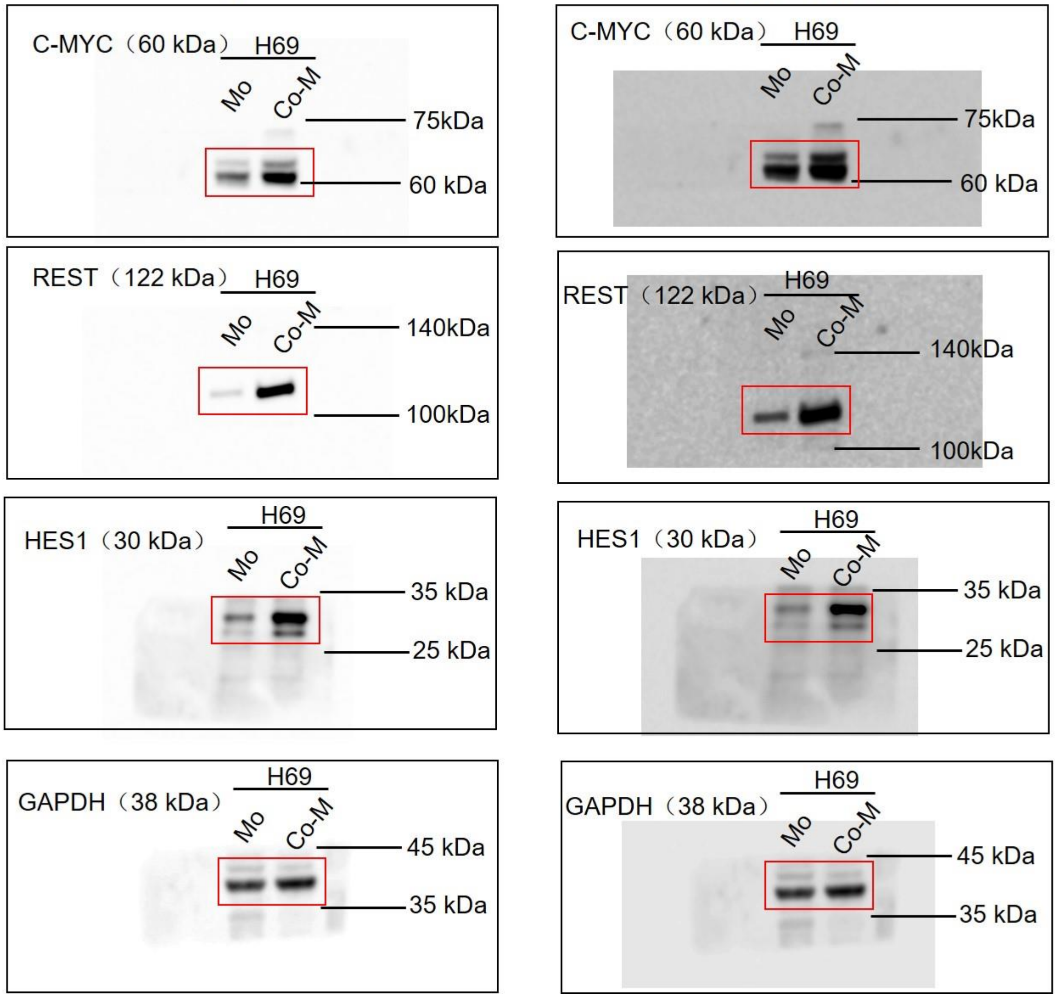

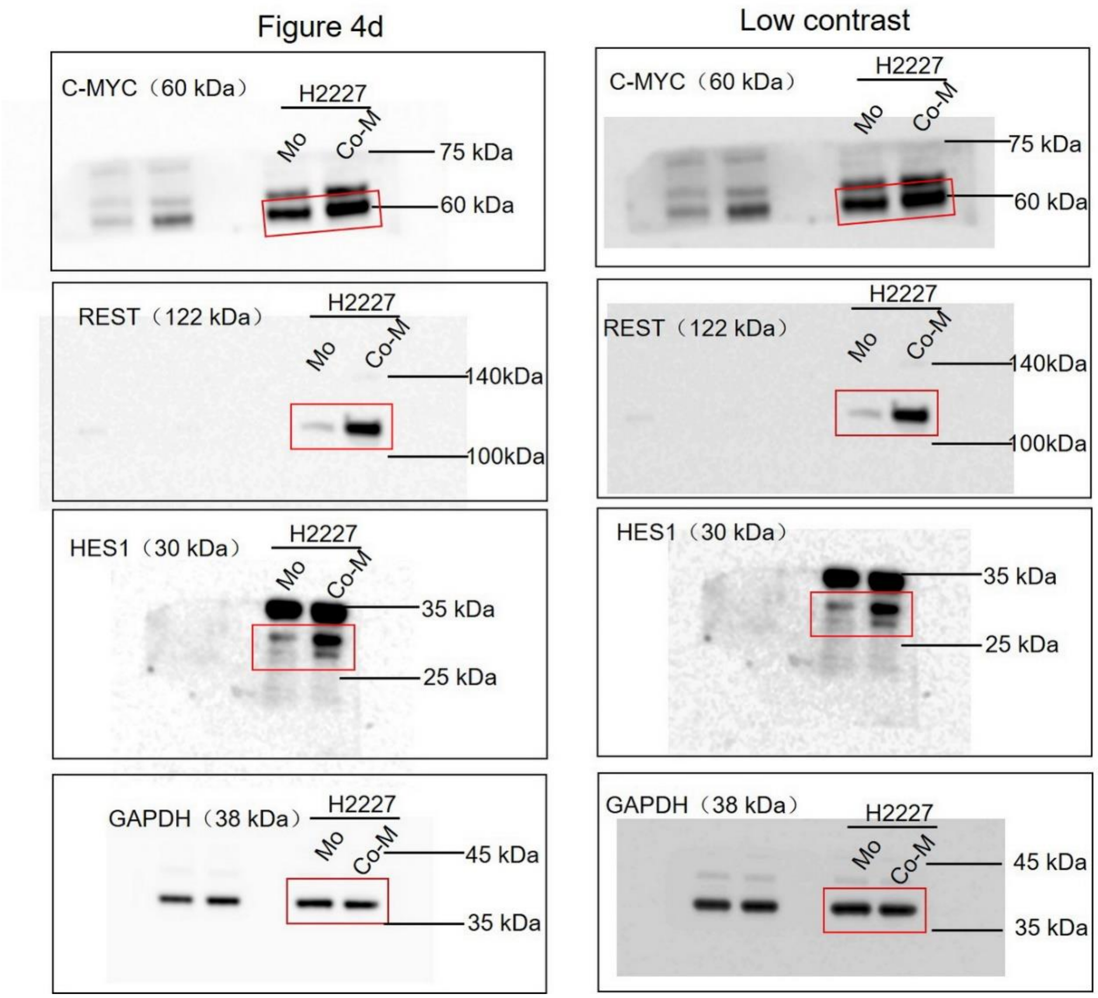
Figure 4d
Low contrast
C-MYC（60 kDa）
H2227
Mo
Co-M
75 kDa
60 kDa
REST（122 kDa）
140kDa
100kDa
HES1（30 kDa）
35 kDa
25 kDa
GAPDH（38 kDa）
45 kDa
35 kDa

Figure 4d   Low contrast

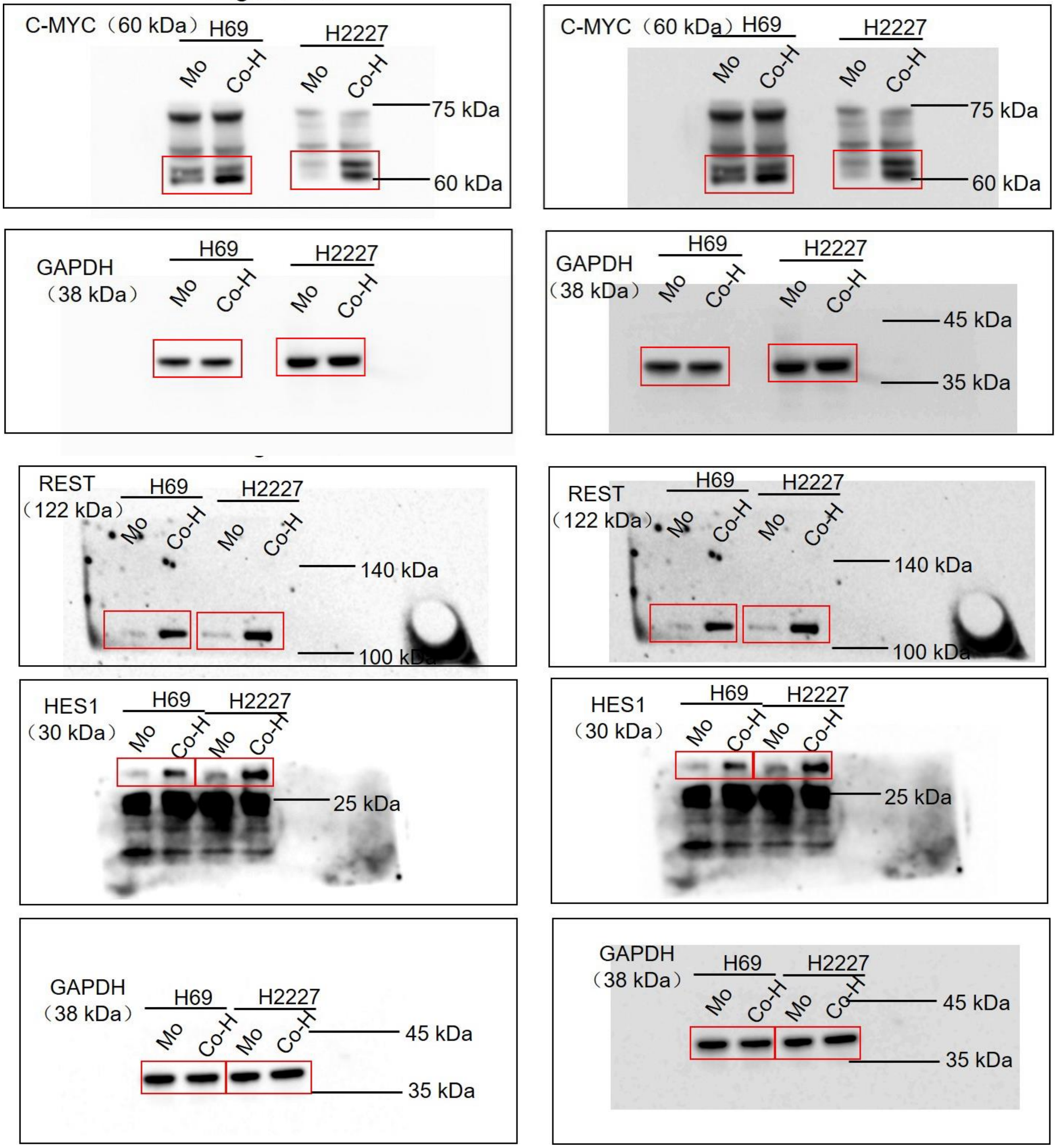

Figure 6a

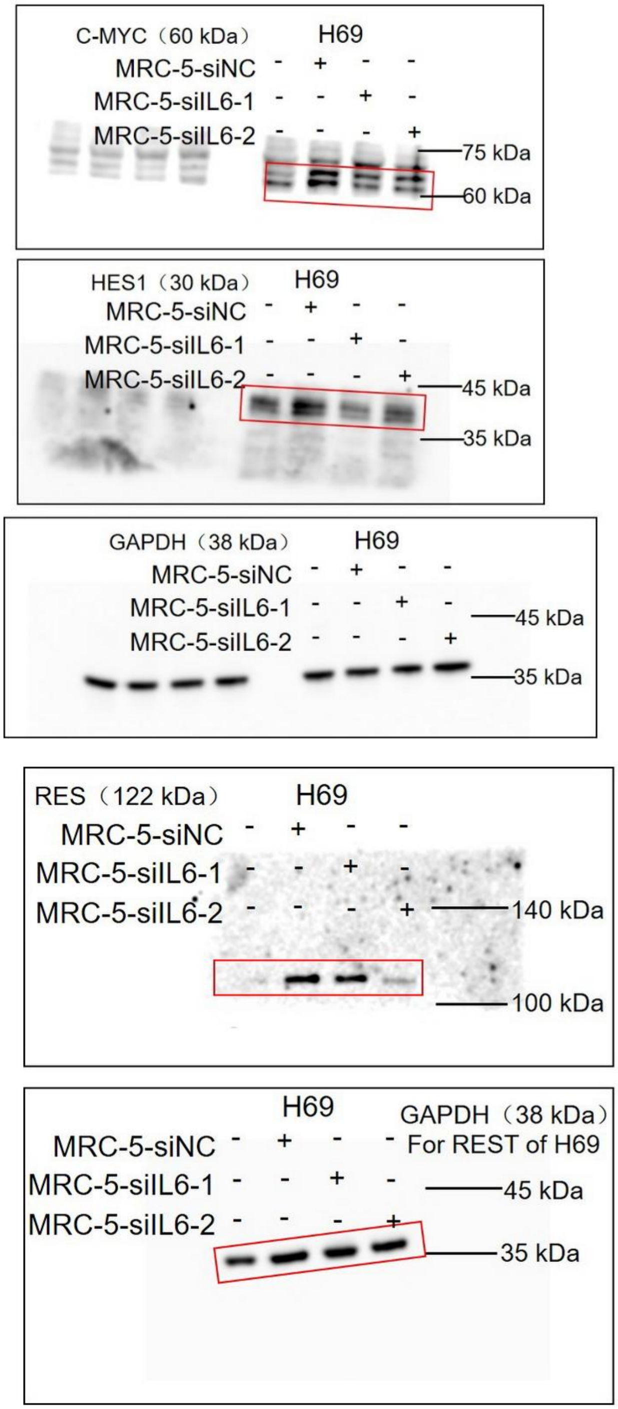

Low contrast

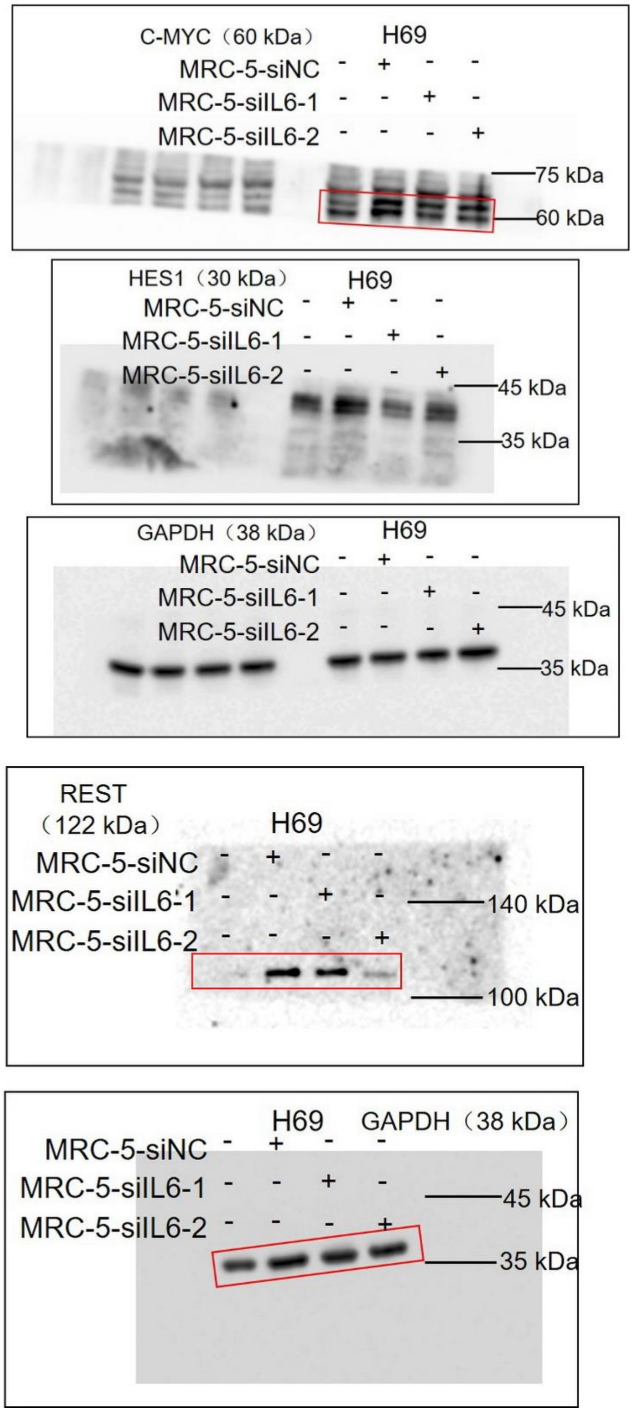

Figure 6a

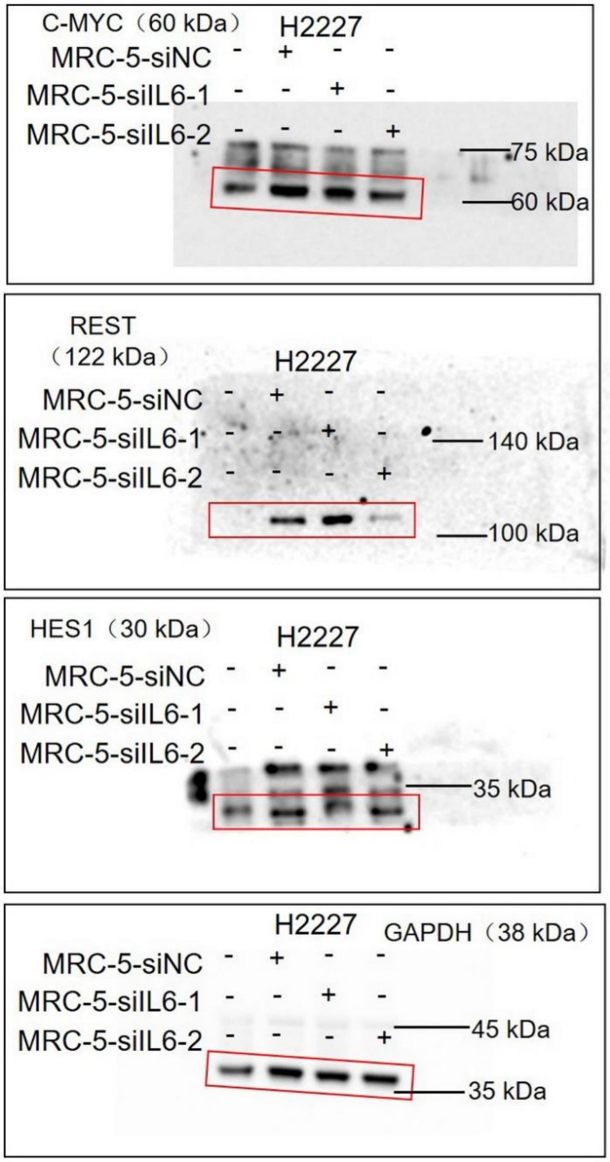

Low contrast

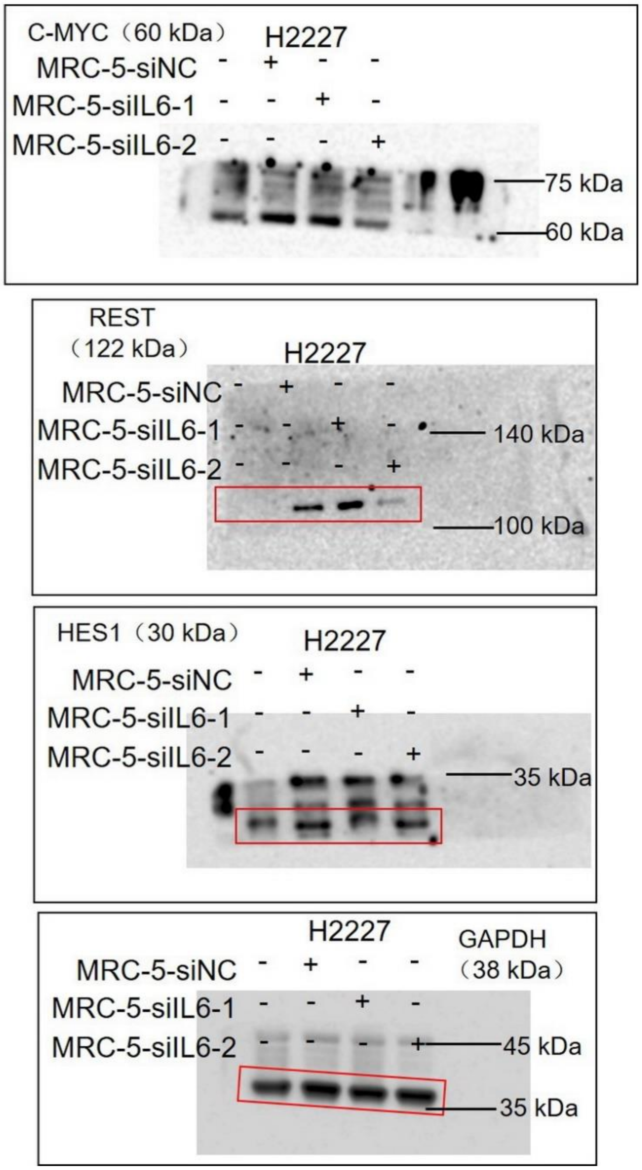

Figure 6b

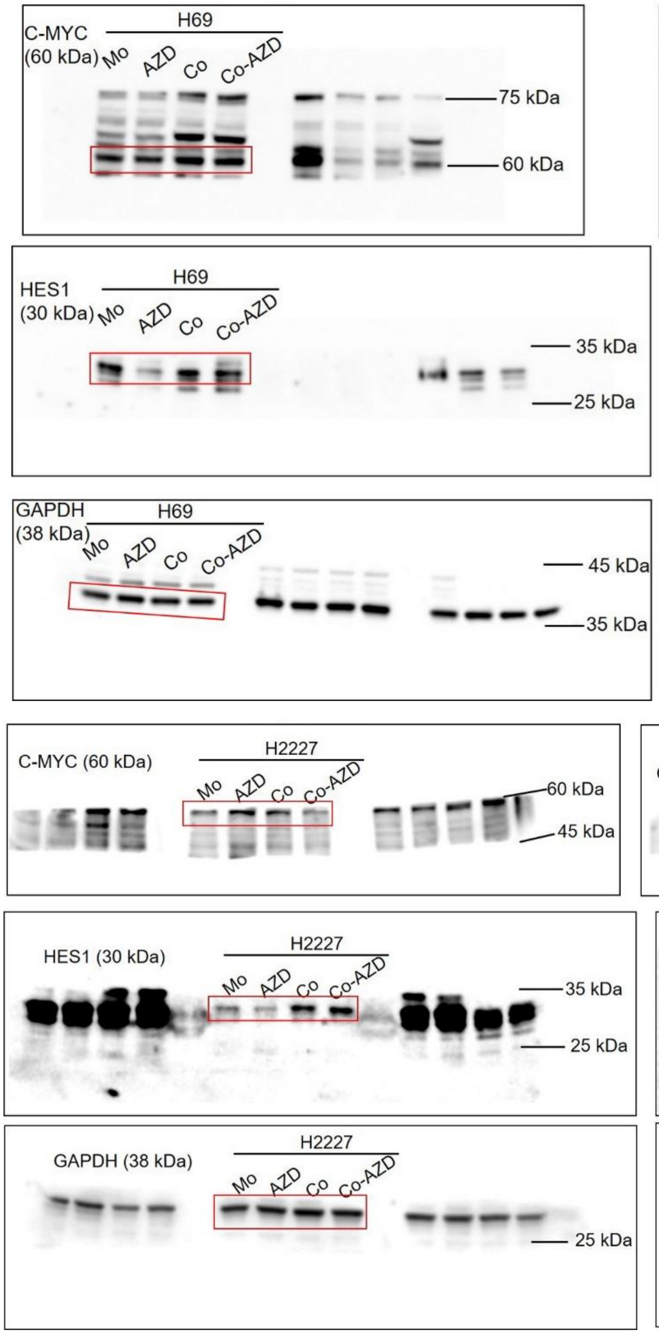

Low contrast

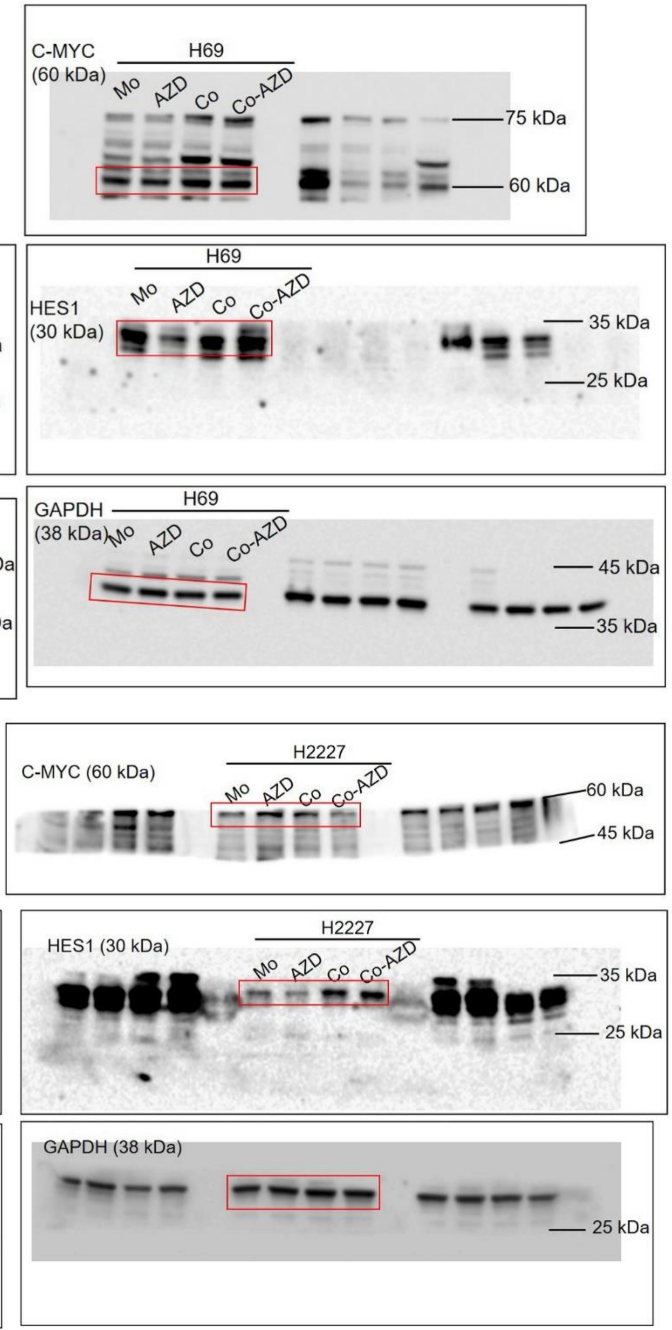

Figure 6e

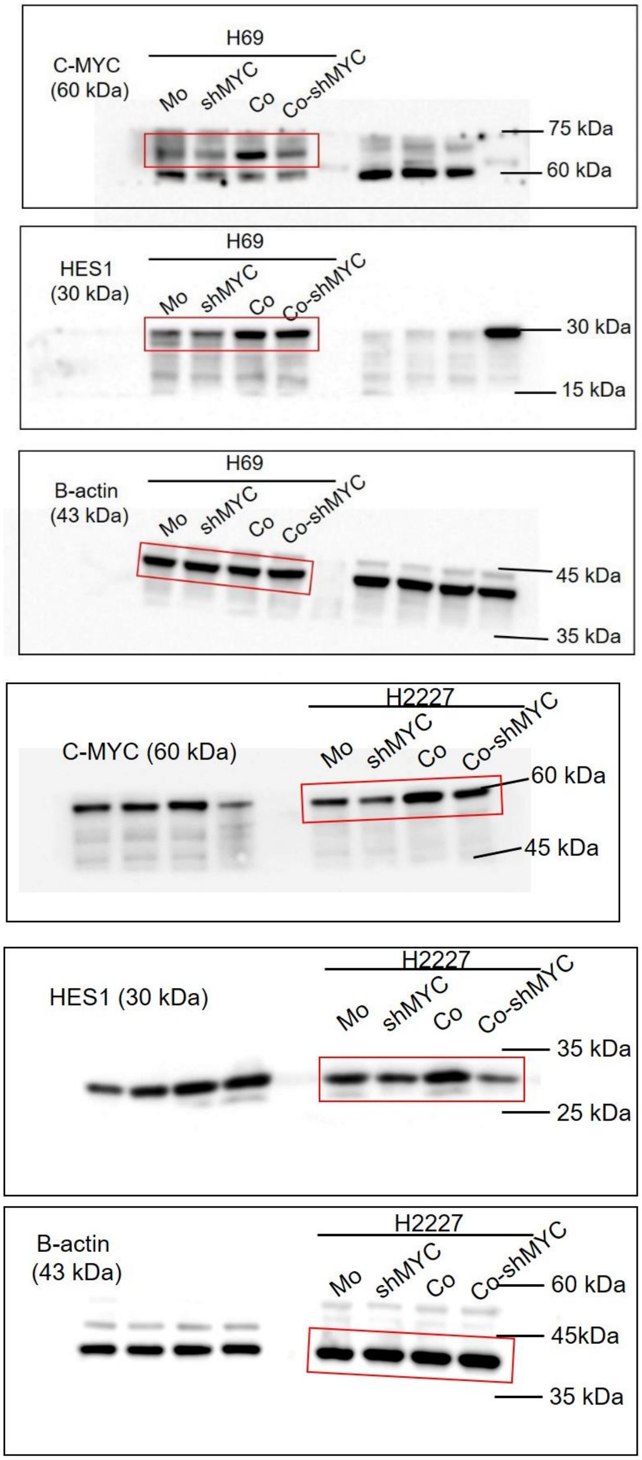

Low contrast

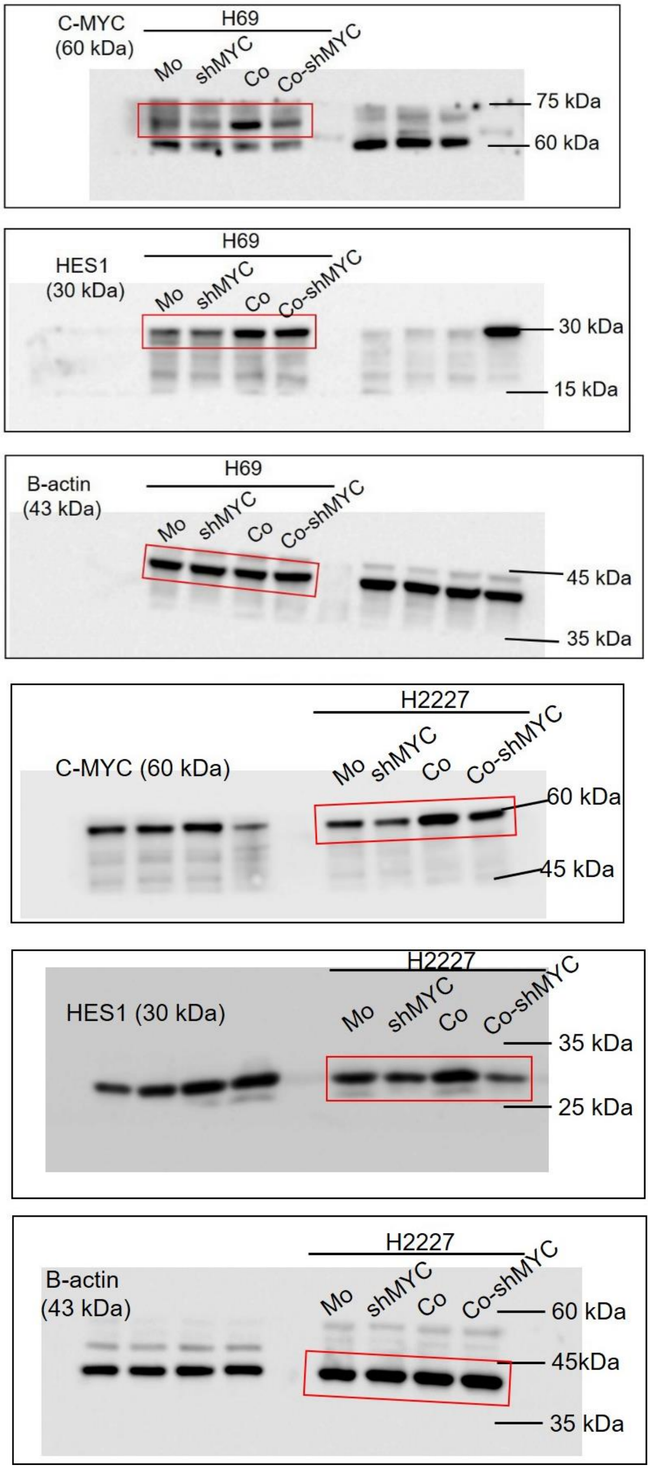

Figure S2a

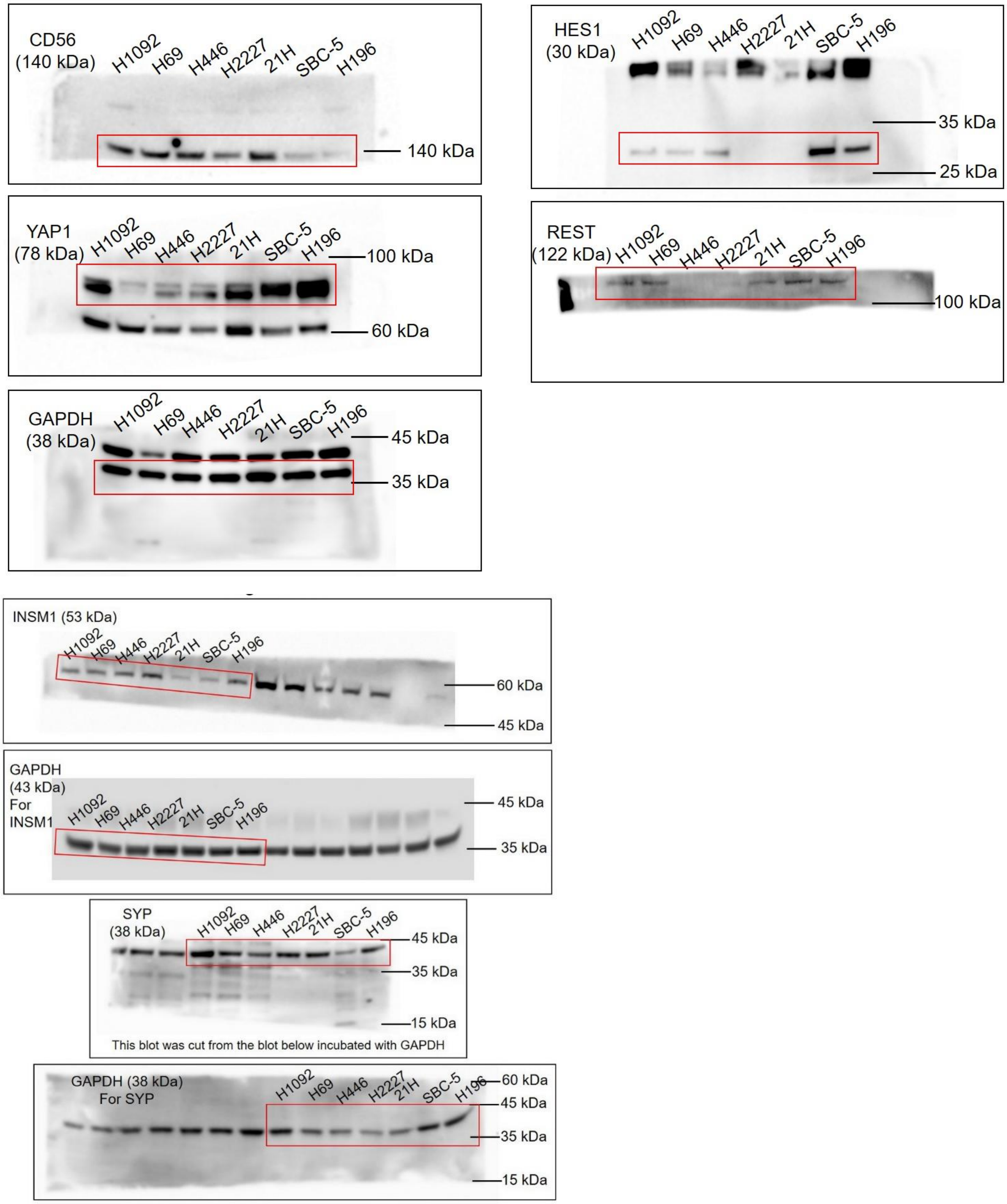
CD56 (140 kDa)
H1092 H69 H446 H2227 21H SBC-5 H196
140 kDa
HES1 (30 kDa)
H1092 H69 H446 H2227 21H SBC-5 H196
35 kDa
25 kDa
YAP1 (78 kDa)
H1092 H69 H446 H2227 21H SBC-5 H196
100 kDa
60 kDa
REST (122 kDa)
H1092 H69 H446 H2227 21H SBC-5 H196
100 kDa
GAPDH (38 kDa)
H1092 H69 H446 H2227 21H SBC-5 H196
45 kDa
35 kDa
INSM1 (53 kDa)
H1092 H69 H446 H2227 21H SBC-5 H196
60 kDa
45 kDa
GAPDH (43 kDa) For INSM1
H1092 H69 H446 H2227 21H SBC-5 H196
45 kDa
35 kDa
SYP (38 kDa)
H1092 H69 H446 H2227 21H SBC-5 H196
45 kDa
35 kDa
15 kDa
This blot was cut from the blot below incubated with GAPDH
GAPDH (38 kDa) For SYP
H1092 H69 H446 H2227 21H SBC-5 H196
60 kDa
45 kDa
35 kDa
15 kDa

Figure S2e

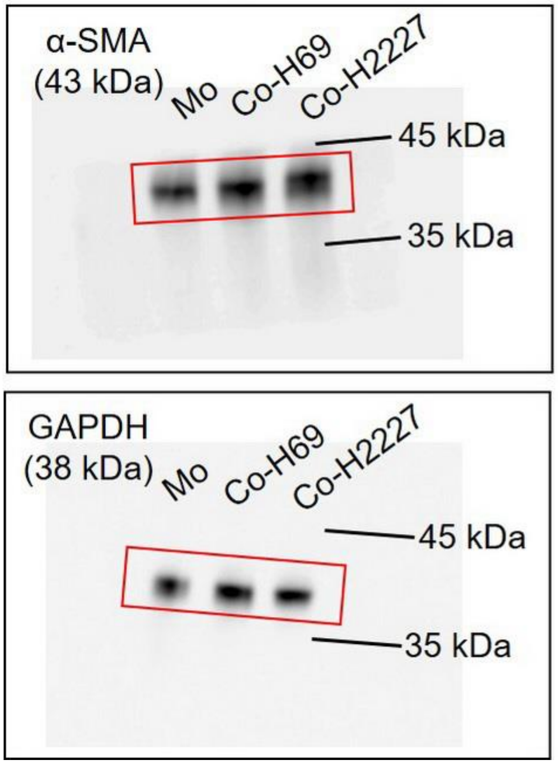

Low contrast

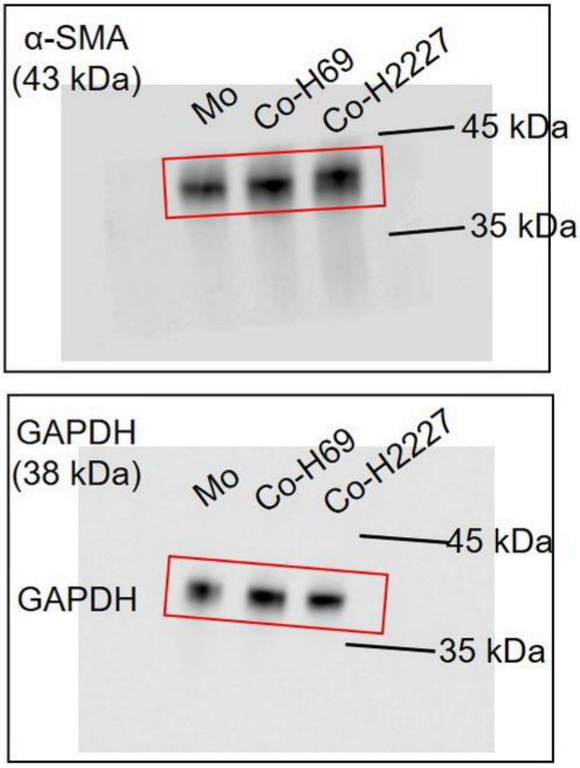

Replicates for western blots

Replicates for Figure 3c

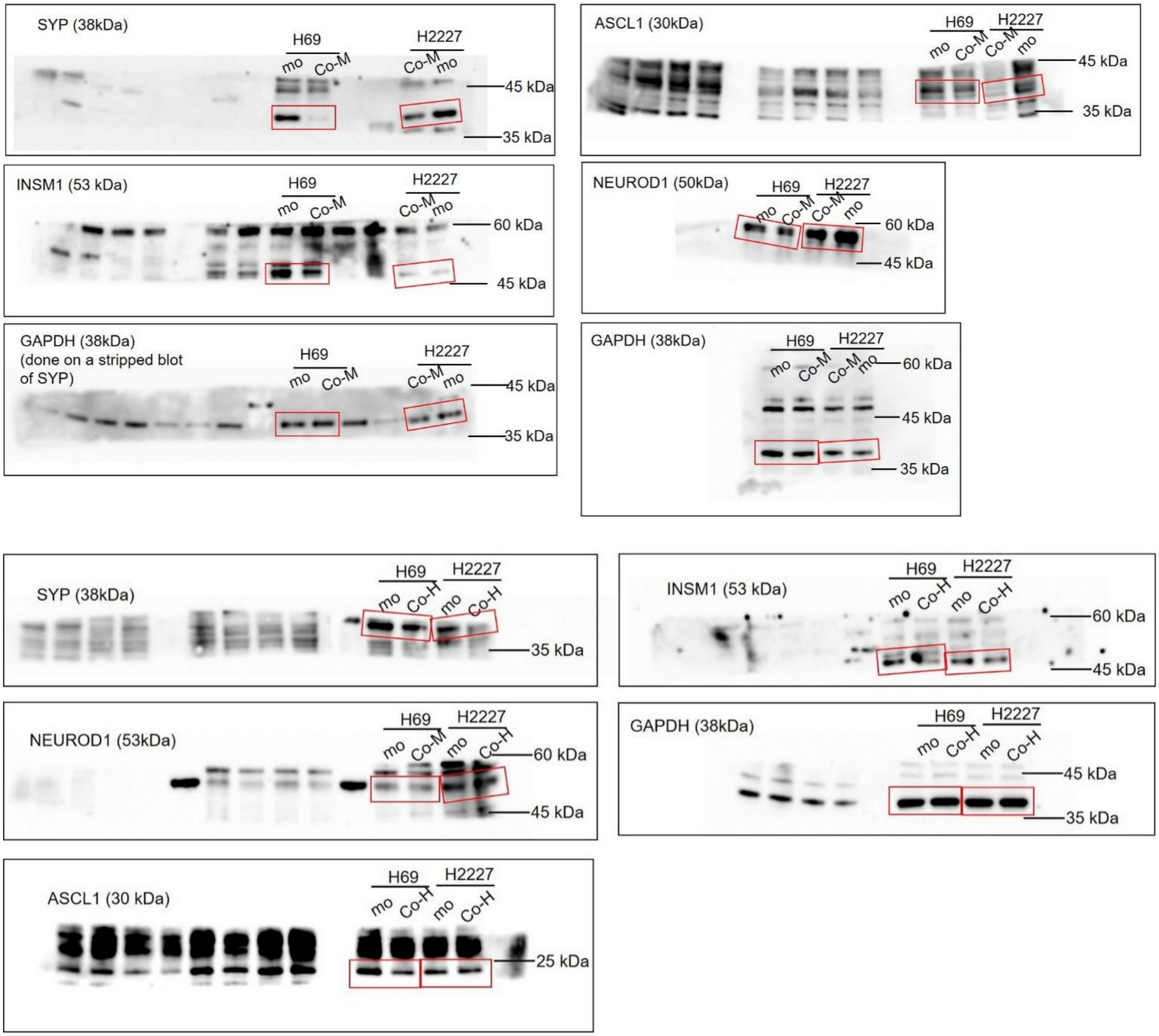

Replicates for Figure 3g

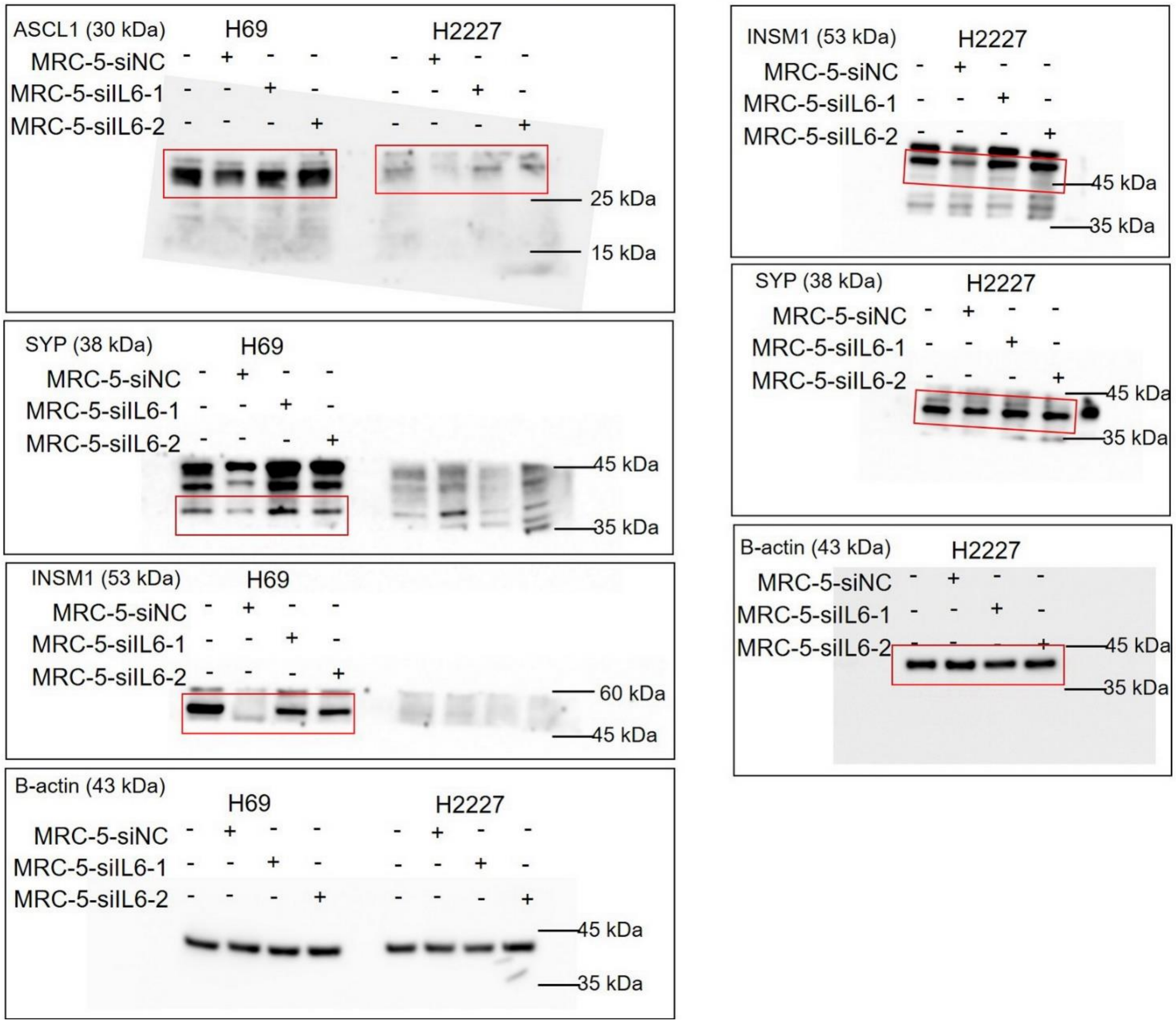

Replicates for Figure 4c

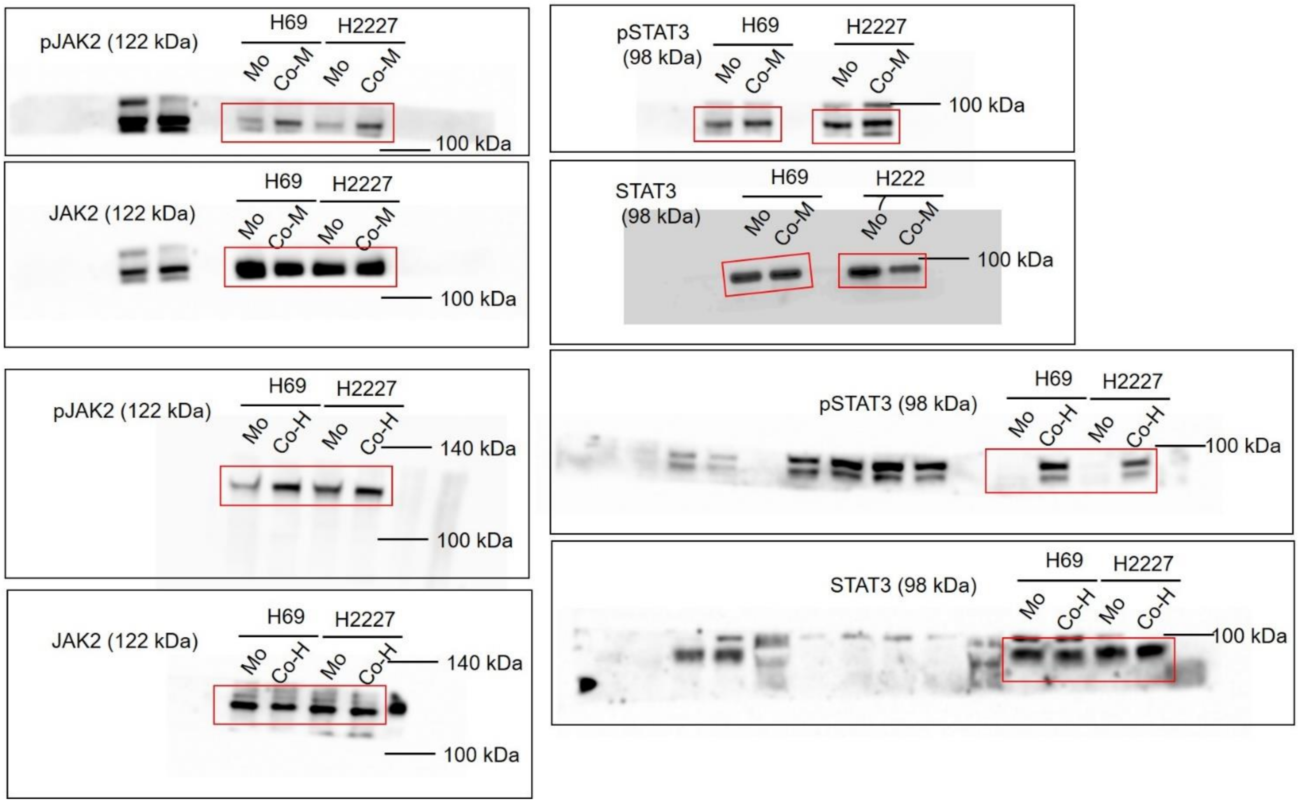

Replicates for Figure 4d

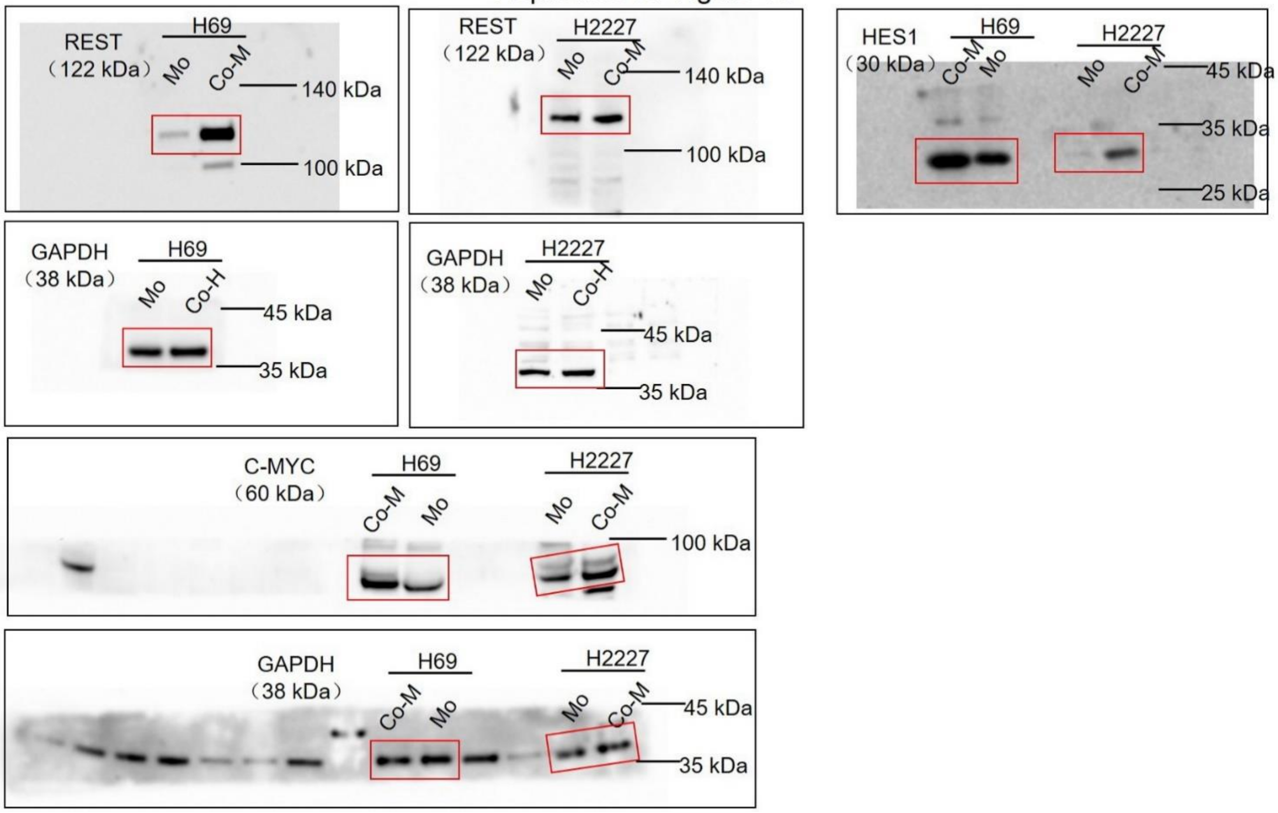

Replicates for Figure 4d

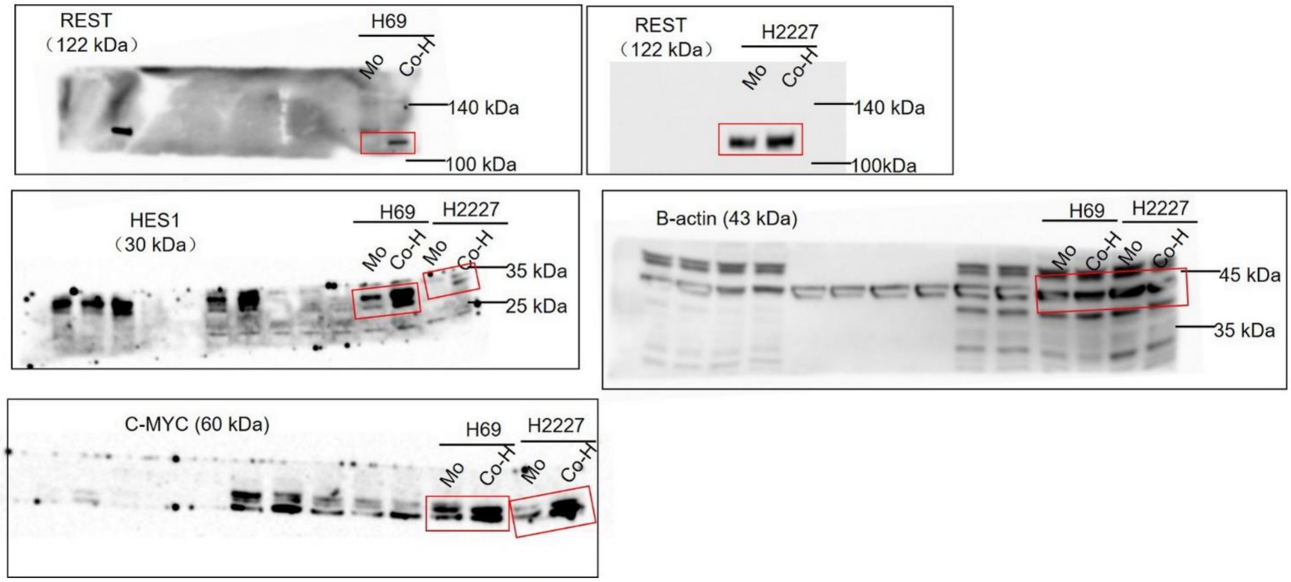

Replicates for Figure 6a

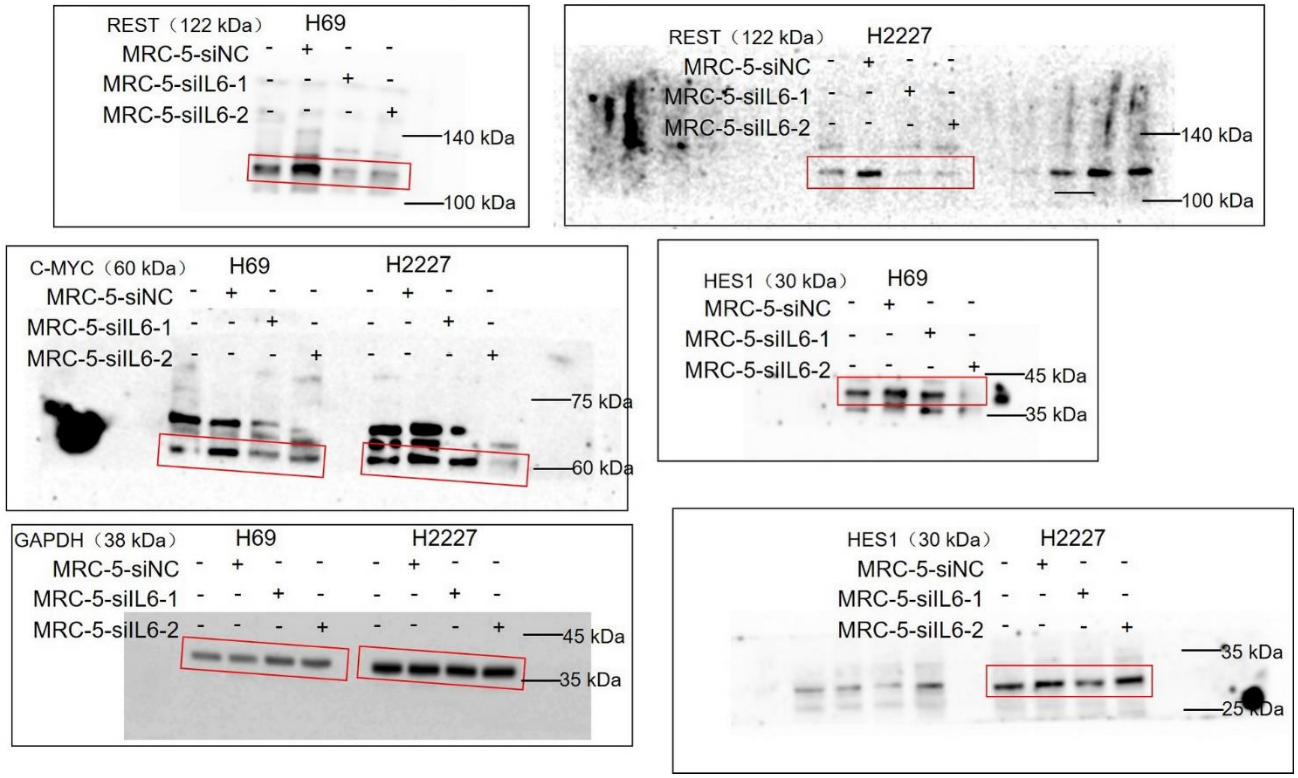

Replicates for Figure 6b

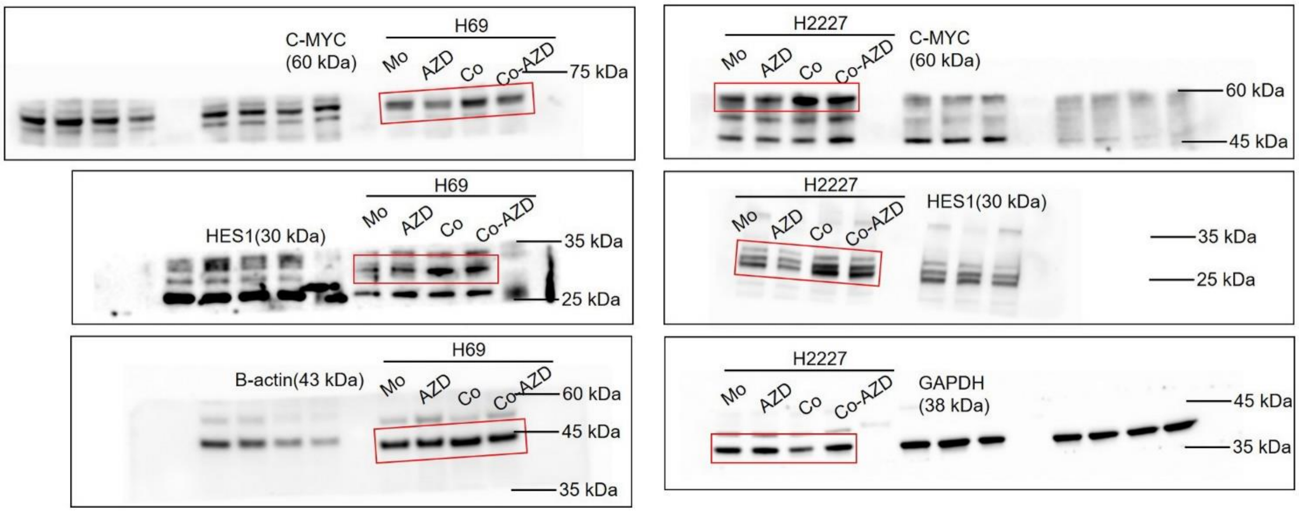

Replicates for Figure 6e

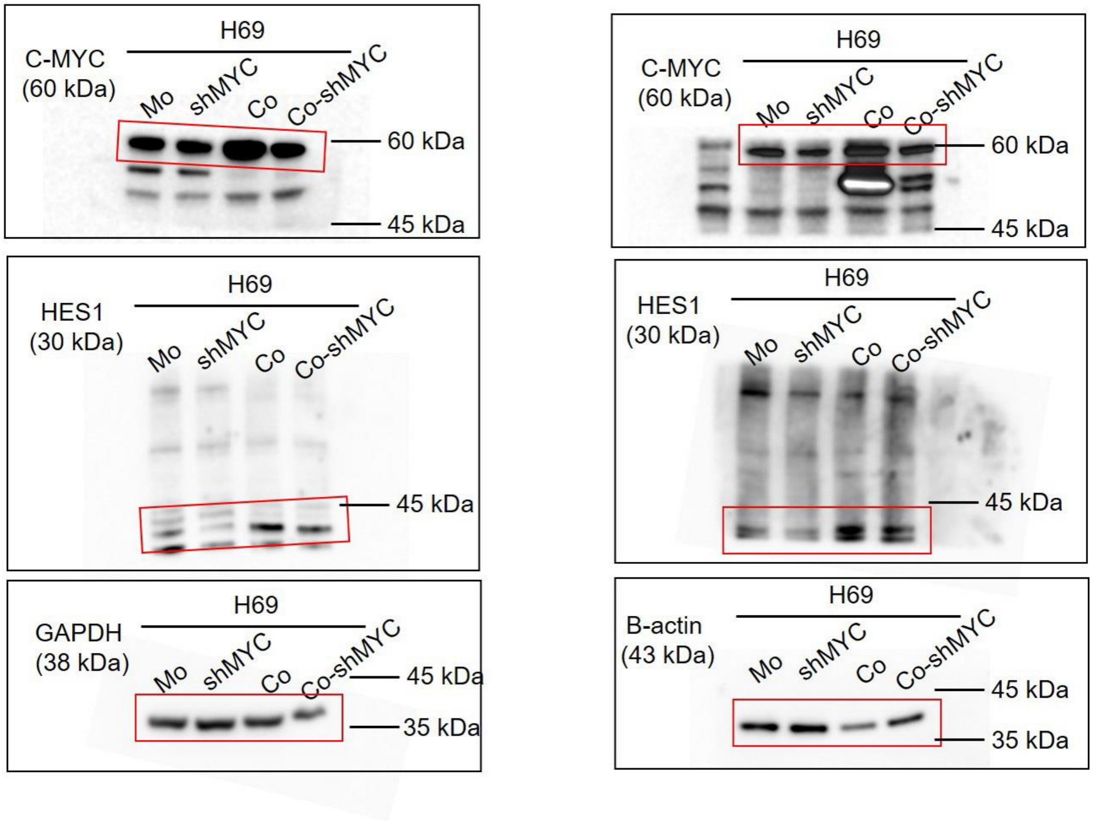

Replicates (fuller-length, original, unprocessed blots)

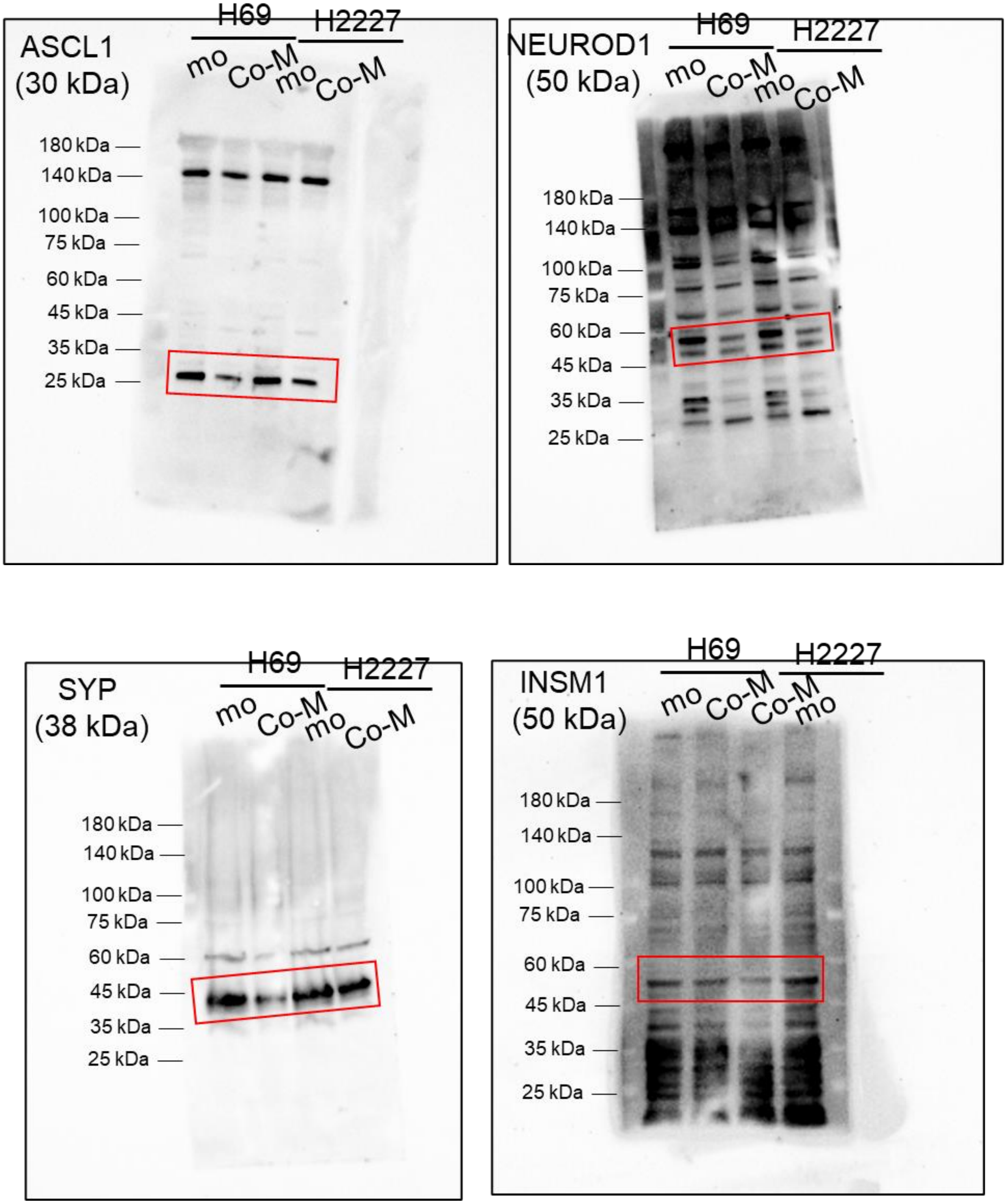

Replicates (fuller-length, original, unprocessed blots)

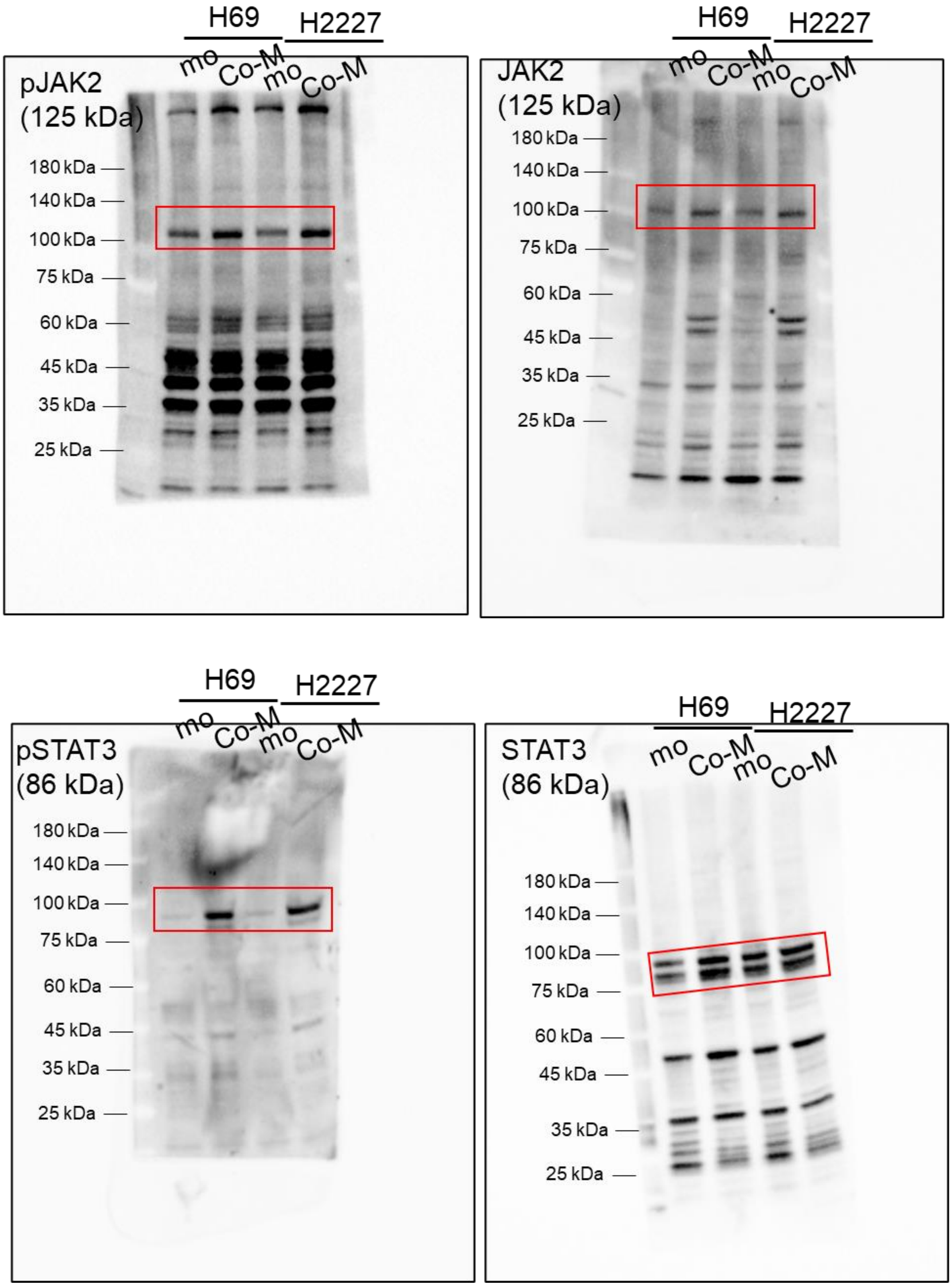

Replicates (fuller-length, original, unprocessed blots)

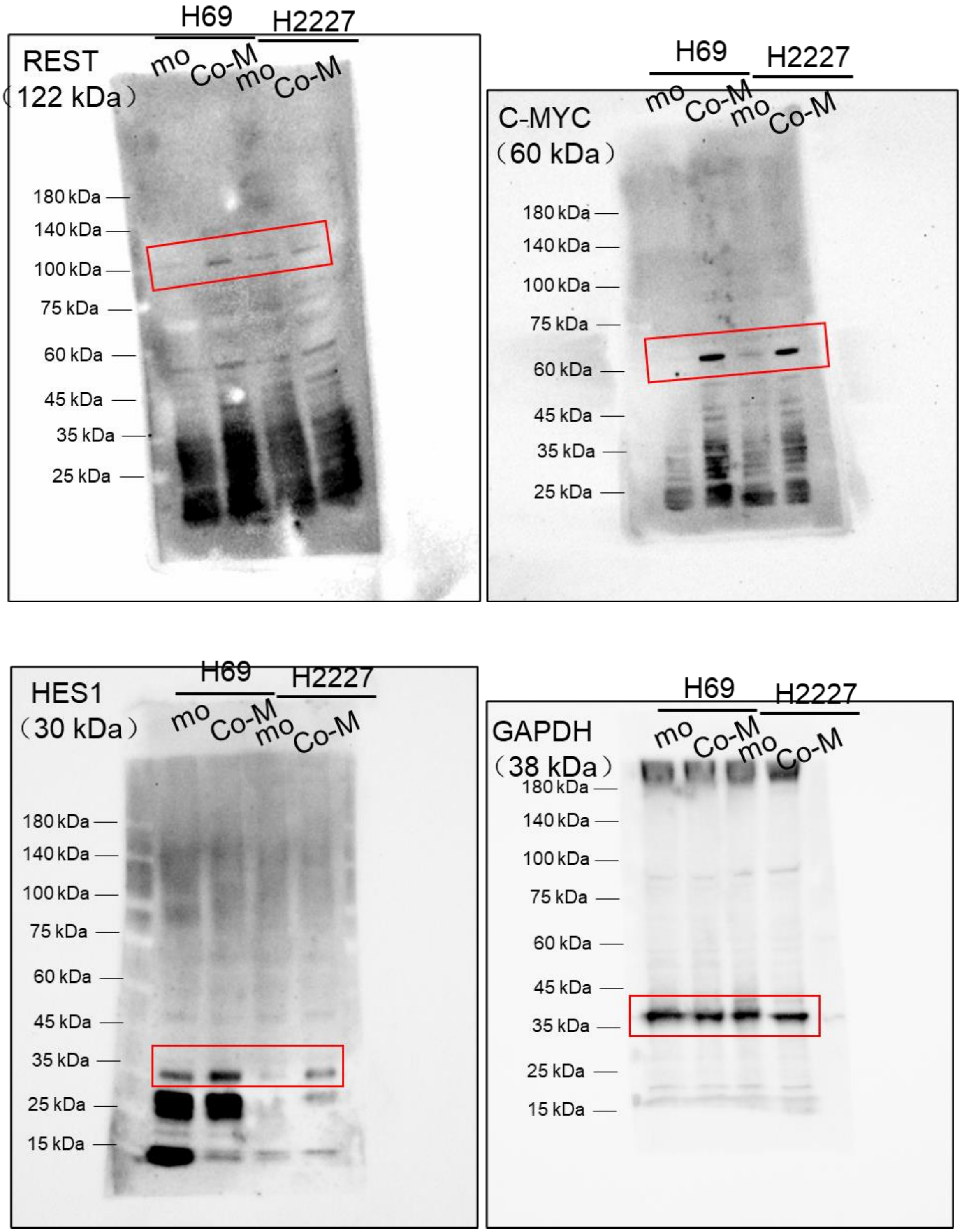

Supplement: Supplementary file 2 — Supplementary Information. [file 41598_2024_52687_MOESM2_ESM.pdf]
